# GSK3β‐Regulated Lipolysis is Required for Histone Acetylation and Decidualization in Early Pregnancy

**DOI:** 10.1002/advs.202514291

**Published:** 2025-11-09

**Authors:** Peiran Wang, Yedong Tang, Xueling Zhao, Yu Ni, Hualan Zhou, Enhao Zhang, Gaizhen Li, Han Cai, Yinan Wang, James R. Woodgett, Wenbo Deng, Haibin Wang, Zhongxian Lu, Haili Bao, Shuangbo Kong

**Affiliations:** ^1^ State Key Laboratory of Vaccines for Infectious Diseases Xiang An Biomedicine Laboratory School of Pharmaceutical Sciences Xiamen University Xiamen Fujian 361102 China; ^2^ Fujian Provincial Key Laboratory of Reproductive Health Research Department of Obstetrics and Gynecology The First Affiliated Hospital of Xiamen University School of Medicine Xiamen University Xiamen Fujian 361102 China; ^3^ Lunenfeld‐Tanenbaum Research Institute Sinai Health and Department of Medical Biophysics University of Toronto Toronto Ontario M5G 1X5 Canada; ^4^ State Key Laboratory of Vaccines for Infectious Diseases Xiang An Biomedicine Laboratory School of Medicine Xiamen University Xiamen Fujian 361102 China; ^5^ Fujian Provincial Key Laboratory of Innovative Drug Target Research School of Pharmaceutical Sciences Xiamen University Xiamen Fujian 361005 China

**Keywords:** decidualization, GSK3β, histone acetylation, Lipid droplet, lipolysis

## Abstract

Decidualization, a highly programmed differentiation process of the uterine stroma, is characterized by significant biochemical remodeling and is essential for pregnancy. However, the functions and molecular mechanisms of lipid metabolism during decidualization remain poorly understood. In this study, a dynamic process of lipid droplet synthesis and degradation is observed during decidual progression, and GSK3 is identified as a potential regulator for lipolysis. Specifically, lipolysis is inhibited in uterine *Gsk3b* knockout mice, leading to impaired terminal differentiation of decidual cells. Mechanistically, GSK3β promots phosphorylation‐dependent lysosomal degradation of RNF213, which permits the localization of adipose triglyceride lipase (ATGL) on lipid droplets, thereby facilitating lipolysis. Furthermore, fatty acids released from lipolysis enter the mitochondria to undergo β‐oxidation and produce acetyl‐CoA. The inhibition of lipolysis caused by GSK3β deficiency leads to a reduction in acetyl‐CoA levels, which in turn epigenetically affects gene transcription through histone acetylation. This study provided evidence for the regulation of dynamic lipid metabolism in vivo, and its influences on gene transcription for decidualization, which emphasized the critical role of metabolic modulation in uteri during early pregnancy.

## Graphical abstract

Uterine *Gsk3b* knockout impairs decidual cell terminal differentiation by causing lipid droplet accumulation. GSK3β phosphorylates RNF213 to promote its lysosomal degradation, thereby enhancing lipolysis. The released fatty acids undergo β‐oxidation to generate acetyl‐CoA, which modulates histone acetylation and regulates decidual cell terminal differentiation‐related gene expression.

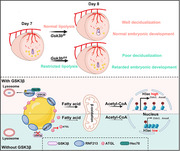

## 1 Introduction

The highly programmed transformation of the endometrium into decidua, known as decidualization, is a key event in early mammalian pregnancy.^[^ ^1^ ^]^ In mice, following embryo implantation, endometrial stromal cells near the embryo undergo complex molecular reprogramming and differentiate into morphologically and functionally distinct secretory decidual cells.^[^ ^2^ ^]^ The decidualization process was mainly regulated by the endocrine hormones and signals from the implanted embryo.^[^ ^3^ ^]^ Fully developed decidual cells provide nutritional support and an immunotolerant microenvironment for peri‐implantation embryo development before the formation of the mature placenta.^[^ ^4^ ^]^ Abnormal decidualization often leads to severe pregnancy defects, such as recurrent miscarriage. However, only limited progress has been made on the molecular regulatory mechanisms of decidualization.

The decidualization process is accompanied by significant metabolic reprogramming to meet the energy and material demands. Several studies have confirmed that glucose metabolism plays a critical role in the decidualization process in both rodents and humans. There is a lactate shuttle between decidual cells and surrounding undifferentiated stromal cells, and disrupting glycolysis or lactate flux impairs decidual formation.^[^ ^5^ ^]^ A group of decidual cells with Gamt expression, defined as nourishing DSCs, accumulates glycogen granules.^[^ ^6^ ^]^ For lipid metabolism, it was reported that fatty acids accumulate in decidual cells on days 5 and 6 of pregnancy (day 1 = the day of the positive of vaginal plug).^[^ ^7^ ^]^ Some of these fatty acids may be used for phospholipid synthesis, but most are metabolized into triglycerides stored in lipid droplets. Fatty acid oxidation is also suggested to be crucial during decidualization, since Cpt1a knockdown impairs decidualization in vitro.^[^ ^8^, ^9^ ^]^ The increase in aquaporin 7 (AQP7) during uterine decidualization correlates with heightened glycerol accumulation in the uterus and an upregulation of glycerol kinase expression.^[^ ^10^ ^]^ The sphingolipid metabolic pathway is also activated during decidualization, and the de novo synthesis of sphingolipids has been shown to be essential for decidualization.^[^ ^11^ ^]^ These metabolic changes support rapid cell proliferation and differentiation, providing essential nutrients and protection for the embryo, thus ensuring a successful pregnancy. These findings highlight the importance and complexity of metabolic regulation, especially lipid metabolism during decidualization. However, how the anabolism and catabolism of lipids are timely regulated during in vivo decidualization still remains elusive.

Our previous findings demonstrated that Iipoprotein lipase (LPL) secreted by primary trophoblast giant cells plays a critical role in lipid droplet accumulation in decidual cells.^[^ ^12^ ^]^ In the current study, we observed the rapid synthesis and degradation of lipids during the progression of decidualization. Focusing on lipid droplet degradation in decidual cells, small‐molecule inhibitor screening identified GSK3 as a potential target molecule regulating lipolysis during decidualization. GSK3β is one critical member in the GSK3 family, and its deletion causes embryo lethality.^[^ ^13^ ^]^ It is a classical protein‐serine kinase that phosphorylates over 100 proteins, regulating many fundamental biological processes, including proliferation and metabolism.^[^ ^14^ ^]^ In a mouse model with uterine‐specific deletion of GSK3β, we observed that decidual cells lacking GSK3β displayed abnormal accumulation of lipid droplets due to inhibited lipolysis. Mechanically, GSK3β was found to interact with and phosphorylate RNF213 to promote its degradation via the lysosomal pathway, and this facilitated the localization of the lipase ATGL onto lipid droplets to mobilize the stored lipid. In reproductive biology, metabolic states can profoundly influence cell fate decisions through epigenetic regulation. Recent studies have revealed that during trophoblast differentiation, glucose metabolism is reduced to a basal level, yet this residual glycolytic activity is crucial for producing acetyl‐CoA that maintains histone acetylation. Such histone acetylation activates key genes involved in trophoblast syncytialization and placental hormone production, thereby linking cellular metabolism to the epigenetic control of placental development and function.^[^ ^15^ ^]^ Furthermore, we demonstrate that free fatty acids derived from the hydrolysis of lipid droplets undergo β‐oxidation to supply acetyl‐CoA. This metabolic pathway is vital for regulating histone acetylation modifications, which, in turn, epigenetically affect transcription of genes associated with decidualization.

## 2 Results

### 2.1 Lipolysis may be Regulated by GSK3β During Decidualization

**Figure 1:**
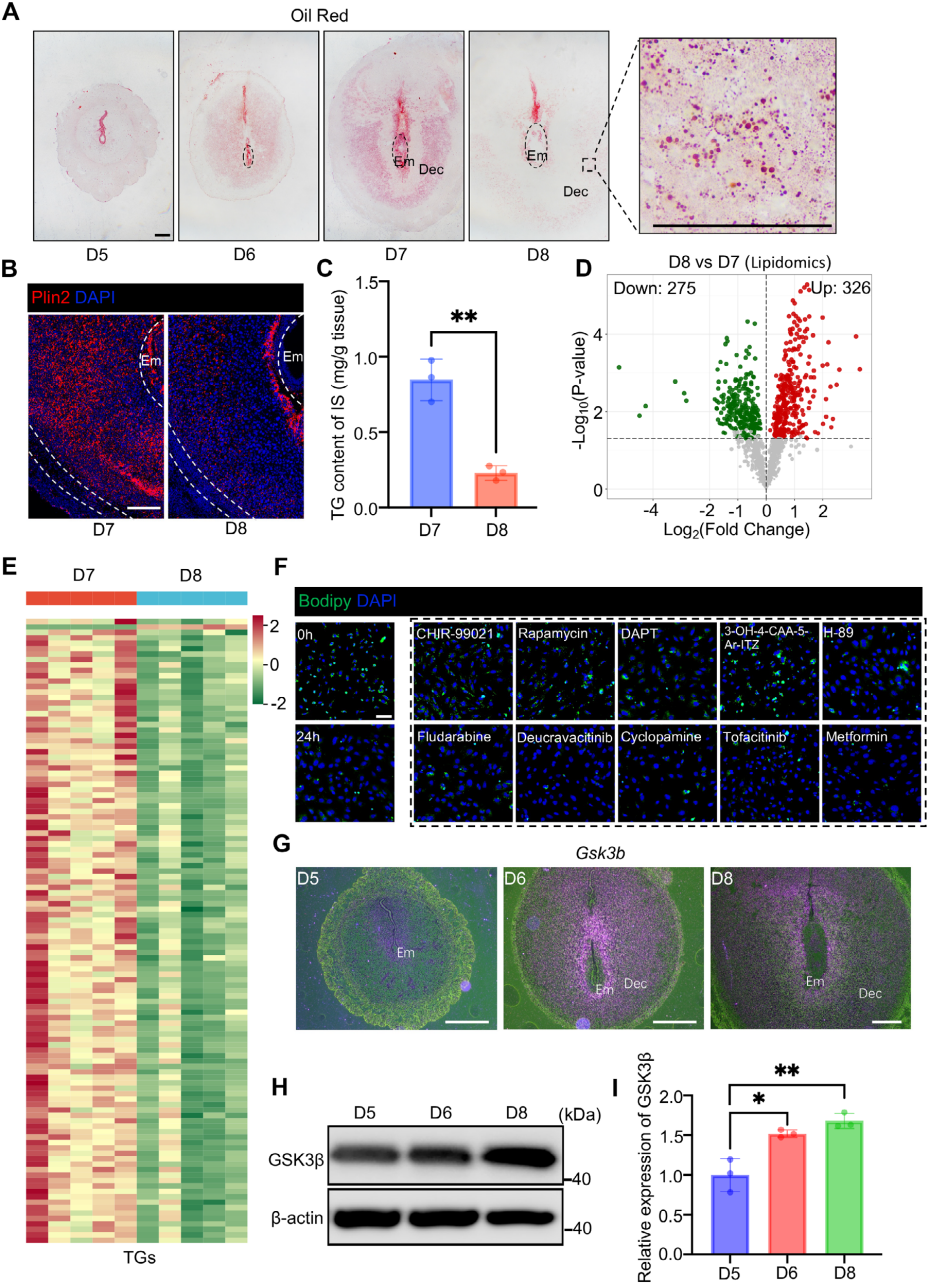
GSK3β may regulate lipolysis during decidualization. A) Oil red staining of lipid droplets in wild type uterine implantation sites on days 5, 6, 7, and 8. Em, embryo, Dec, decidual cell. Scale bars: 200 µm. B) Immunofluorescence analysis of Plin2 in wild‐type uterine implantation sites on day 7 and day 8. Scale bars: 200 µm. C) TG content assay of wild type (*N* = 3 animals) uterine implantation sites on days 7 and 8. Data represent the mean ± SEM. Two‐tailed unpaired Student's *t*‐test, ^**^ *p* = 0.0019. D) Volcano plot showing the significantly changed lipid species between day 7 and day 8 decidual tissues. E) Heatmap showing the relative abundance of TGs between day 7 and day 8 decidual tissues. F) Immunofluorescence analysis of Bodipy in the primary decidual cells treated with different inhibitors. The dashed box represents the screening results after treatment for 24 h. Scale bars: 50 µm. G) In situ hybridization of *Gsk3b* in wild‐type uteri on days 5, 6, and 8 of pregnancy. Scale bars: 500 µm. H) Western blot analysis of GSK3β in wild‐type uteri on days 5, 6, and 8 of pregnancy. I) Quantification of data in Figure H (*N* = 3 animals), data represent the mean ± SEM. Two‐tailed unpaired Student's *t*‐test, ^*^ *p* = 0.0132, ^**^ *p* = 0.0067.

Decidualization is accompanied by significant metabolic reprogramming. We observed a dynamic process of lipid droplet accumulation from day 5 to day 7 as revealed by oil red staining, followed by rapid degradation when decidualization progressed to day 8 (**Figure** **1**A). Both immunofluorescence staining of lipid droplet coating protein Plin2 and quantitative triglyceride measurements demonstrated rapid lipid droplet breakdown during terminal differentiation of decidual cells from day 7 to day 8 (Figure 1B,C; Figure S1A, Supporting Information). Lipidomics also revealed significant lipid remodeling during the terminal differentiation of decidual cells (Figure 1D). The major changes were observed in triglycerides (TGs), showing a general downregulation across species with different side chain compositions (Figure 1E; Table S1, Supporting Information). Our previous report has uncovered the embryo's signal to induce lipid accumulation in decidua.^[^ ^12^ ^]^ In this study, we focus on investigating the regulation of lipid droplet degradation. A small scale of targeted small molecule inhibitors was applied to the primary decidual cells isolated from day 7 mouse uteri with abundant lipid droplets. In the control group, the lipolysis was observed in decidual cell after cultured for 24 h (Figure 1F), which is consistent with in vivo observation. Since the decidualization was a developmental‐like process, several developmental pathways, including the Wnt, Notch, and TGFβ, were pharmacological targeted to explore their potential regulation for this lipolysis. Several positive targets, including the GSK3, BMP2, and Notch, were uncovered to regulate the lipolysis (Figure 1F; Figure S1B, Supporting Information). BMP2 and Notch signals have been reported to regulate the decidualization,^[^ ^16^, ^17^ ^]^ so the GSK3β was chosen for our following study. The GSK3 family consists of two members, GSK3α and GSK3β. There was no reproductive phenotype in *Gsk3a* knockout female mice, but *Gsk3b* knockout was embryo lethality,^[^ ^13^ ^]^ suggesting the potential role of *Gsk3b* for this lipolysis during decidualization.

Next, we investigated the expression of *Gsk3b* in the peri‐implantation uterus. In situ hybridization (ISH)  data revealed that *Gsk3b* exhibited widespread expression during decidualization. On day 5, *Gsk3b* transcripts were detected in proliferating stromal cells surrounding the implanting blastocyst. Accompanying the initiation of decidualization on day 6 until the decidua was fully developed on day 8, the level of expression of *Gsk3b* was maintained throughout the entire uterine stromal cell population, implying a close association between *Gsk3b* and the process of decidualization (Figure 1G; Figure S1C, Supporting Information). Immunohistochemical revealed extensive cytoplasmic expression of GSK3β protein. (Figure S1D, Supporting Information). Moreover, immunoblot data show that the protein level of GSK3β was increased on day 8 when the lipolysis occurred (Figure 1H,I).

### 2.2 Mice with Ablation of Uterine *Gsk3b* Lead to Impaired Lipid Degradation and Subfertility

**Figure 2:**
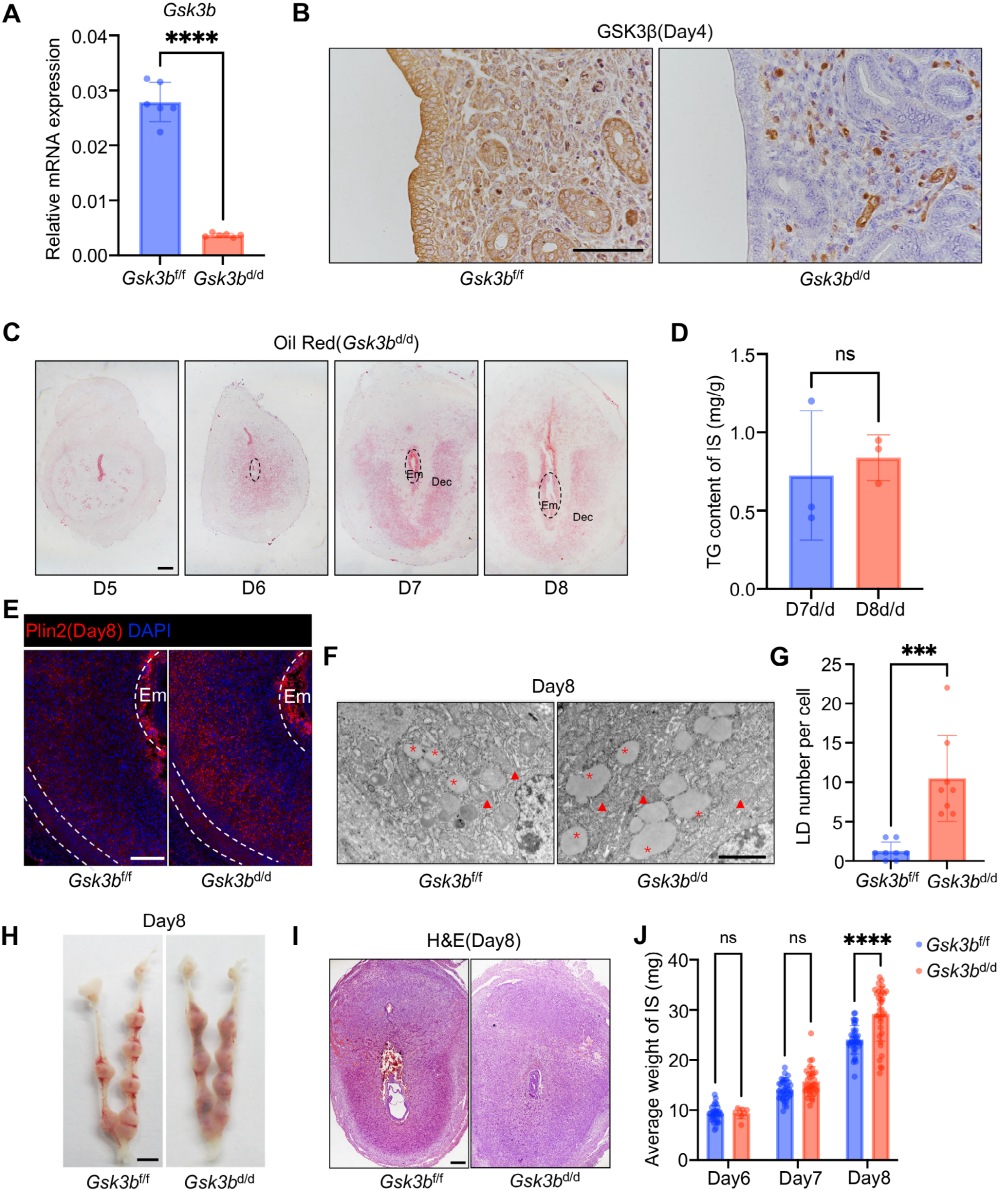
Ablation of uterine *Gsk3b* leads to impaired lipolysis and abnormal decidualization. A) Quantitative real‐time PCR analysis of *Gsk3b* mRNA levels in *Gsk3b* ^f/f^ (*N* = 6 animals) and *Gsk3b* ^d/d^ (*N* = 6 animals) implantation sites on day 8. The values are normalized to *Gapdh* and indicated as the mean ± SEM. Two‐tailed unpaired Student's t‐test, ^****^ *p* < 0.0001. B) Immunohistochemical analysis of GSK3β in *Gsk3b* ^f/f^ and *Gsk3b* ^d/d^ uteri on day 4. C) Oil red staining of lipid droplets in *Gsk3b* ^d/d^ implantation sites on days 5,6,7, and 8. Em, embryo, Dec, decidua cell. Scale bars: 200 µm. D) TG content assay of *Gsk3b* ^d/d^ (*N* = 3 animals) implantation sites on days 7 and 8. Data represent the mean ± SEM. Two‐tailed unpaired Student's t‐test. ns, not significant. E) Immunofluorescence analysis of Plin2 in *Gsk3b* ^f/f^ and *Gsk3b* ^d/d^ implantation sites on day 8. Scale bars: 200 µm. F) Transmission electron microscopy (TEM) analysis of decidual cells in *Gsk3b* ^f/f^ and *Gsk3b* ^d/d^ uteri on day 8 of pregnancy. Red asterisk, lipid droplets, Red arrow, mitochondria. G) Average number of lipid droplets of decidual cells in *Gsk3b* ^f/f^ (*N* = 8 cells) and *Gsk3b* ^d/d^ (*N* = 8 cells) mice. Data represent the mean ± SEM. Two‐tailed unpaired Student's *t*‐test, ^***^ *p* = 0.0004. H) Representative images of day 8 pregnant uteri in *Gsk3b* ^f/f^ and *Gsk3b* ^d/d^ mice. Scale bar: 5 mm. I) Histology of embryo development in *Gsk3b* ^f/f^ and *Gsk3b* ^d/d^ implantation sites on day 8. Scale bar: 200 µm. J) The average weight of the implantation sites in *Gsk3b* ^f/f^ and *Gsk3b* ^d/d^ mice on days 6, 7, and 8. Data represent the mean ± SEM. Two‐tailed unpaired Student's *t*‐test, ^****^ *p* < 0.0001, ns, not significant.

To elucidate the functional role of uterine *Gsk3b* in pregnancy, we generated mice with uterine deletion of *Gsk3b* (*Gsk3b* ^d/d^) by crossing *Gsk3b* floxed mice (*Gsk3b* ^f/f^) with Pgr‐Cre mice (*Pgr* ^Cre/+^).^[^ ^18^ ^]^ We first assessed the efficacy of *Gsk3b* deletion at both mRNA and protein levels, revealing successful *Gsk3b* depletion within the uterus (**Figure** **2**A,B; Figure S2A, Supporting Information). To explore whether *Gsk3b* deletion affects female fertility, we mated *Gsk3b* ^f/f^ and *Gsk3b* ^d/d^ female mice with fertile wild‐type males. The litter size of *Gsk3b* ^d/d^ mice was lower compared to their *Gsk3b* ^f/f^ littermates (Figure S2B, Supporting Information). These findings indicated that uterine *Gsk3b* plays an essential role in ensuring a successful pregnancy.

In mice, decidualization was induced by the embryo implantation, and when the implantation status was checked on day 5, it was noticed that the number of implantation sites was comparable between the *Gsk3b* ^f/f^ and *Gsk3b* ^d/d^ mice (Figure S2C,D, Supporting Information), and the embryo attachment reaction revealed by COX2 was also occurred normally (Figure S2E,F, Supporting Information).^[^ ^19^ ^]^ In addition, the expression levels of FOXO1 and PGR in the epithelium showed no significant differences, indicating that the loss of *Gsk3b* does not affect embryo implantation (Figure S2G–J, Supporting Information).^[^ ^20^ ^]^ On day 6, when the decidual reaction initiates, the morphology of the decidual bulge and the number of implantation sites were similar between *Gsk3b* ^f/f^ and *Gsk3b* ^d/d^ mice (Figure S2K,L, Supporting Information). When the lipid droplet distribution was examined, it was found that lipid could accumulate normally prior to day 7, but failed to undergo proper degradation from day 7 to day 8 in the decidual cells of *Gsk3b* ^d/d^ mice (Figure 2C; Figure S2M, Supporting Information), which confirmed our in vitro observation. Quantitative triglyceride assays revealed accumulation of lipid in decidual cells of *Gsk3b* ^d/d^ mice on day 8 (Figure 2D).  Both immunofluorescence staining of Plin2 and transmission electron microscopy analyses consistently demonstrated suppressed lipolysis during decidualization in *Gsk3b* ^d/d^ mice (Figure 2E–G; Figure S2N, Supporting Information). Compared to *Gsk3b* ^f/f^ mice on day 8, *Gsk3b* ^d/d^ mice exhibit retarded embryo development and an abnormal increase in weight of implantation sites (Figure 2H–J). On day 10, pronounced hemorrhage and resorption appeared at the implantation sites in *Gsk3b* ^d/d^ mice (Figure S2O, Supporting Information). In summary, these results indicate that uterine‐specific deletion of *Gsk3b* disrupts lipolysis during decidualization, leading to embryo resorption and miscarriage at mid‐gestation.

### 2.3 Uterine Deletion of *Gsk3b* Results in Defective Terminal Differentiation of Decidual Cells

**Figure 3:**
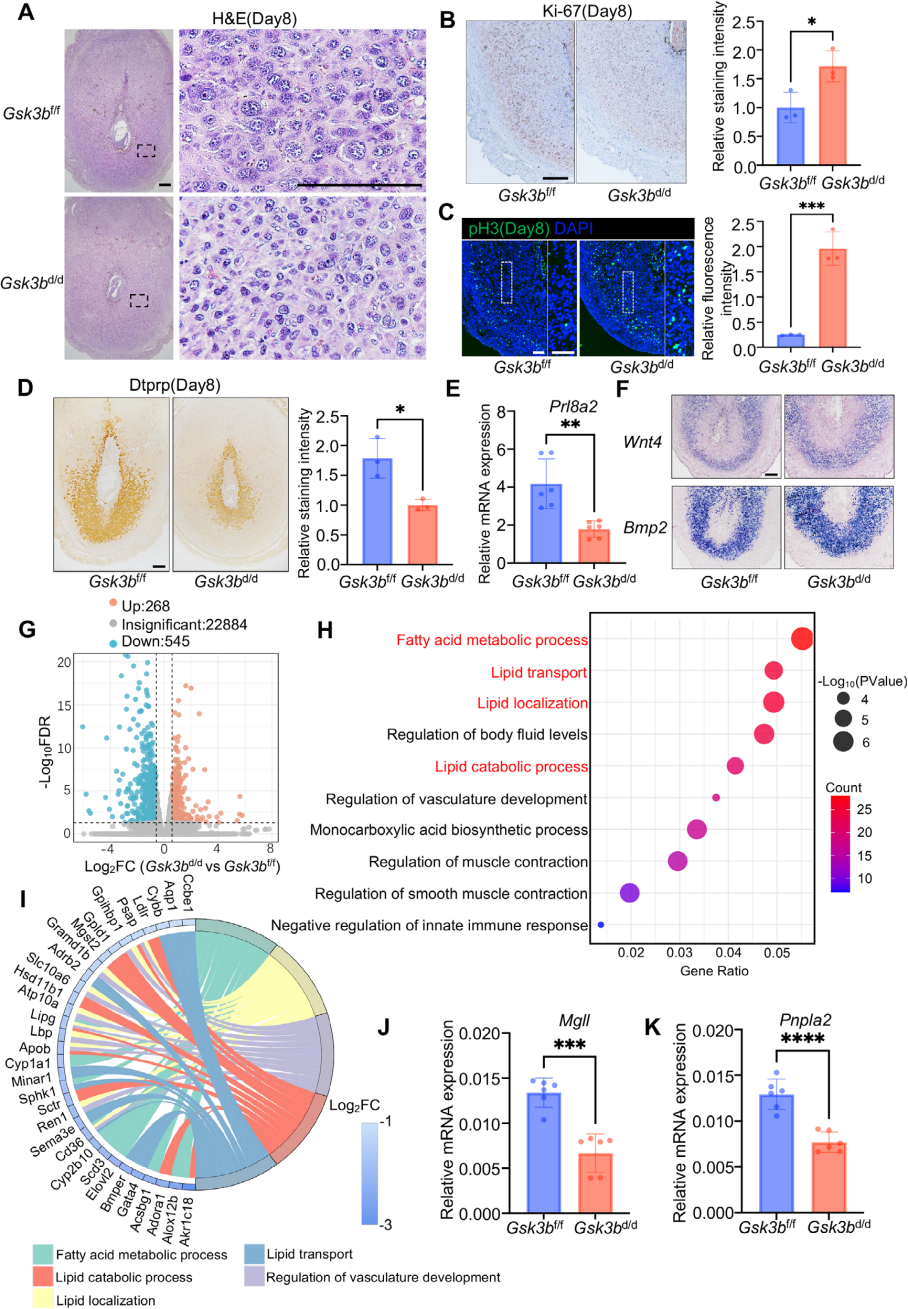
Uterine *Gsk3b* deficiency derails the terminal differentiation of decidual cells. A) Morphometric analysis of *Gsk3b* ^f/f^ and *Gsk3b* ^d/d^ decidual cells on day 8. Scale bars: 200 µm. B) Immunohistochemical analysis of Ki‐67 in *Gsk3b* ^f/f^ and *Gsk3b* ^d/d^ implantation sites on day 8. Scale bars: 200 µm. Quantification of the Ki‐67‐positive area is shown on the right. Data represent the mean ± SEM (*N* = 3 animals). Two‐tailed unpaired Student's *t*‐test, ^*^ *p* = 0.0303. C) Immunofluorescence analysis of pH3 in *Gsk3b* ^f/f^ and *Gsk3b* ^d/d^ implantation sites on day 8. Scale bars: 100 µm. Quantification of the pH3‐positive area is shown on the right. Data represent the mean ± SEM (*N* = 3 animals). Two‐tailed unpaired Student's *t*‐test, ^***^ *p* = 0.0009. D) Immunohistochemical analysis of decidual marker Dtprp in *Gsk3b* ^f/f^ and *Gsk3b* ^d/d^ implantation sites on day 8. Scale bars: 200 µm. Quantification of the Dtprp‐positive area is shown on the right. Data represent the mean ± SEM (*N* = 3 animals). Two‐tailed unpaired Student's *t*‐test, ^*^ *p* = 0.0170. E) Quantitative real‐time PCR analysis of *Prl8a2* mRNA levels in *Gsk3b* ^f/f^ (*N* = 3 animals) and *Gsk3b* ^d/d^ (*N* = 3 animals) implantation sites on day 8. The values are normalized to *Gapdh* and indicated as the mean ± SEM. Two‐tailed unpaired Student's *t*‐test, ^**^ *p* = 0.0017. F) In situ hybridization of *Wnt4* and *Bmp2* in *Gsk3b* ^f/f^ and *Gsk3b* ^d/d^ uteri on day 8. Scale bar: 200 µm. G) Volcano plots showing the differentially expressed genes in *Gsk3b* ^f/f^ and *Gsk3b* ^d/d^ uteri on day 8 of pregnancy. H) Gene Ontology functional analysis for downregulated genes between *Gsk3b* ^f/f^ and *Gsk3b* ^d/d^ using DAVID by Kappa Statistics (*p* < 0.05). I) Enrichment chord diagram displaying biological processes corresponding to downregulated genes between *Gsk3b* ^f/f^ and *Gsk3b* ^d/d^ uteri on day 8 of pregnancy. J,K) Quantitative real‐time PCR analysis of *Mgll* and *Pnpla2* mRNA levels in *Gsk3b* ^f/f^ (*N* = 3 animals) and *Gsk3b* ^d/d^ (*N* = 3 animals) implantation sites on day 8. The values are normalized to *Gapdh* and indicated as the mean ± SEM. Two‐tailed unpaired Student's *t*‐test, ^***^ *p* = 0.0001, ^****^ *p* < 0.0001.

Due to the abnormal decidualization observed on day 8, we investigated the molecular defects in decidual cells in the uteri of *Gsk3b* ^d/d^ mice. Uterine sections on day 8 showed that the morphology of decidual cells in *Gsk3b* ^d/d^ mice differed from that in *Gsk3b* ^f/f^ mice. Mature decidual cells typically undergo endoreplication to form mono or bi‐nucleated enlarged cells. In contrast, decidual cells lacking *Gsk3b* exhibited smaller nuclei. (**Figure** **3**A). The abnormal decidualization can be caused by various factors, such as aberrant proliferation and differentiation of decidual cells or defective vascular development.^[^ ^4^, ^21^ ^]^ We first checked the proliferation status of decidual cells on day 8. Under normal circumstances, fully developed decidual cells are expected to exit the cell cycle. The results indicated that the decidual cells in *Gsk3b* ^d/d^ mice were in a state of excessive proliferation, accompanied by aberrant Ki‐67 and pH3 signals (Figure 3B,C). Furthermore, the decreased expression of Dtprp, a marker of decidualization, indicated insufficient terminal differentiation of decidual cells in *Gsk3b* ^d/d^ mice (Figure 3D,E). Fully differentiated decidual cells in the inner zone of implantation sites gradually lost expression of *Bmp2* and *Wnt4*,^[^ ^16^, ^22^ ^]^ but the uterine expression of *Bmp2* and *Wnt4* in *Gsk3b* ^d/d^ mice is significantly broader than that observed in *Gsk3b* ^f/f^ mice, suggesting hindered terminal differentiation of decidual cells in *Gsk3b* ^d/d^ mice (Figure 3F). Together, these observations provide compelling evidence for defective decidualization in the absence of uterine *Gsk3b*.

To further investigate the molecular mechanism underlying the abnormal terminal differentiation of decidual cells in *Gsk3b* ^d/d^ mice, we isolated the decidual tissues from the uteri of *Gsk3b* ^f/f^ and *Gsk3b* ^d/d^ mice on day 8 and analyzed the gene expression profiles using RNA sequencing (RNAseq). Compared to *Gsk3b* ^f/f^ mice, *Gsk3b* ^d/d^ mice displayed a total of 813 differentially expressed genes, with 268 genes upregulated and 545 genes downregulated (Figure 3G). GO pathway enrichment analysis showed that the downregulated genes were mainly concentrated in lipid metabolism‐related pathways (Figure 3H). We confirmed the downregulated genes related to lipid metabolism, including *Pnpla2* and *Mgll*, both of which were downregulated following *Gsk3b* deletion (Figure 3I–K). These results demonstrated that suppressed lipolysis serves as a potential underlying mechanism for impaired decidualization.

The process of decidualization is regulated by ovarian steroid hormones.^[^ ^1^ ^]^ Since the Pgr‐Cre mouse model could also induce conditional gene deletion in the developing corpus luteum, which produces progesterone, we subsequently examined progesterone synthesis in the ovary. On day 8, there was no significant difference in the localization and expression of the key steroid biosynthetic enzymes, cytochrome P450 cholesterol side‐chain cleavage enzyme (P450scc) or 3β‐hydroxysteroid dehydrogenase (3β‐HSD) between *Gsk3b* ^f/f^ and *Gsk3b* ^d/d^ mice (Figure S3A–C, Supporting Information). Serum progesterone (P4) and 17β‐estradiol (E2) levels were also comparable (Figure S3D,E, Supporting Information). The expression of PGR and its target genes Hand2 and Hoxa10 in decidual cells was unaffected by the knockout of *Gsk3b* on day 8 (Figure S3F–J, Supporting Information). These data suggested that the defective decidualization primarily originated from an autonomous dysfunction of *Gsk3b* in stromal cells.

### 2.4 GSK3β Regulates Lipolysis Through ATGL in a Wnt/β‐Catenin Independent Manner

**Figure 4:**
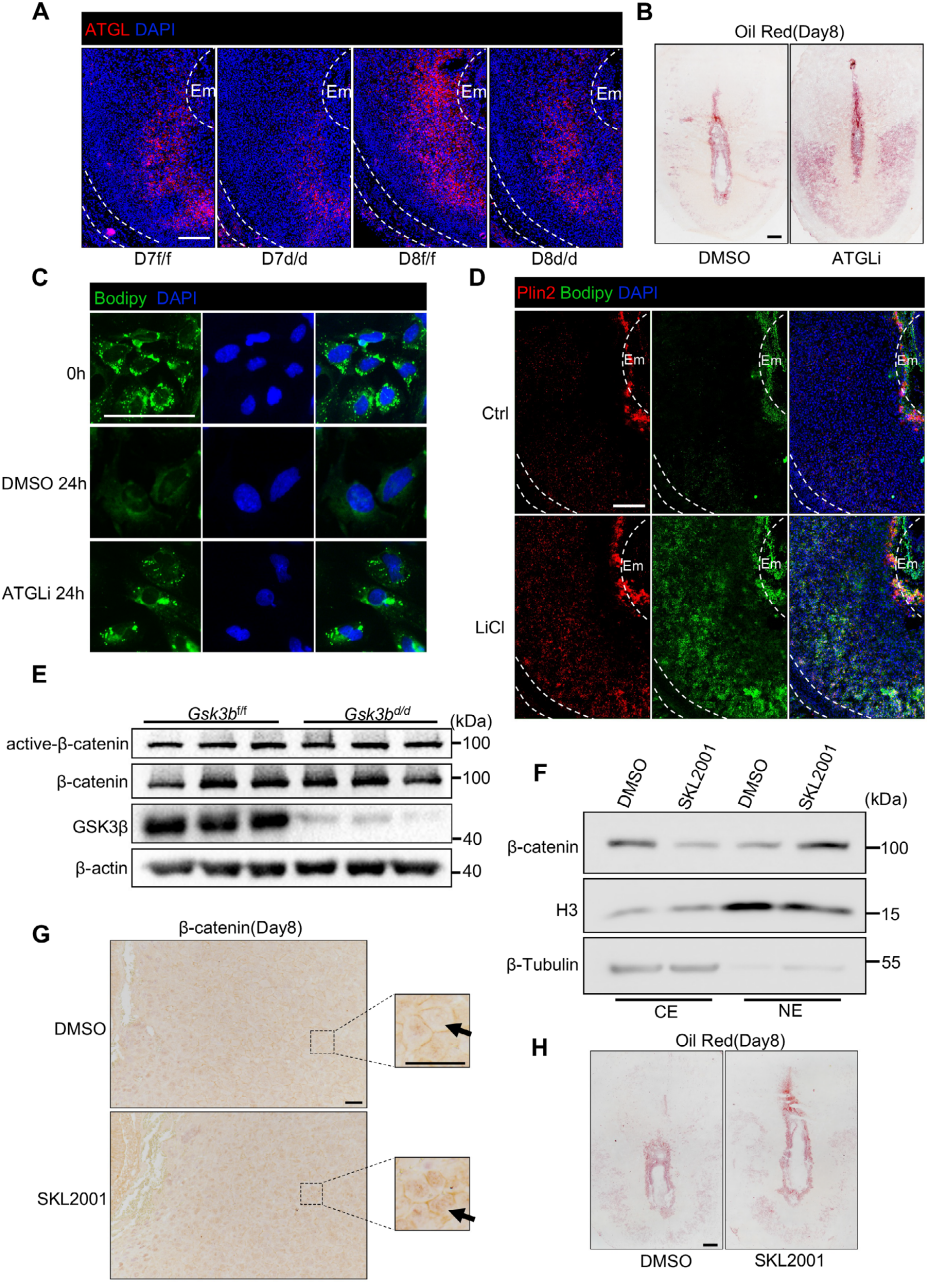
GSK3β may regulate lipolysis through ATGL in a Wnt/β‐catenin independent manner. A) Immunofluorescence analysis of ATGL in *Gsk3b* ^f/f^ and *Gsk3b* ^d/d^ implantation sites on days 7 and 8. Scale bars: 200 µm. B) Oil red staining of lipid droplets in *Gsk3b* ^f/f^ implantation sites treated with DMSO or ATGLi (40 mg kg^−1^) on day 8. Scale bars: 200 µm. C) Immunofluorescence analysis of Bodipy in the primary decidual cells treated with DMSO or ATGLi (20 µm) in 0 or 24 h. Scale bars: 50 µm. D) Immunofluorescence analysis of Plin2 and Bodipy in the implantation sites of mice treated with control or LiCl (150 mg kg^−1^) on day 8. Scale bars: 200 µm. E) Western blot analysis of active‐β‐catenin and β‐catenin in *Gsk3b* ^f/f^ and *Gsk3b* ^d/d^ implantation sites on day 8. F) Western blot analysis of β‐catenin in different cellular fractions from *Gsk3b* ^f/f^ mice implantation sites treated with DMSO or SKL2001 (50 mg kg^−1^) on day 8. G) Immunohistochemical analysis of β‐catenin in *Gsk3b* ^f/f^ and *Gsk3b* ^d/d^ implantation sites treated with DMSO or SKL2001 (50 mg kg^−1^) on day 8. Scale bars: 50 µm. H) Oil red staining of lipid droplets in *Gsk3b* ^f/f^ implantation sites treated with DMSO or SKL2001 (50 mg kg^−1^) on day 8. Scale bars: 200 µm.

Neutral lipolysis involves the coordinated action of ATGL, HSL, and MGL.^[^ ^23^ ^]^ Triglycerides are first hydrolyzed by rate‐limiting lipase ATGL into free fatty acids and diglycerides. Given the crucial role of ATGL in lipolysis, we first examined the expression of uterine ATGL during the lipolysis process. The results showed that from day 7 to day 8, ATGL expression increased in the uteri of *Gsk3b* ^f/f^ mice, but ATGL expression in the uteri of *Gsk3b* ^d/d^ mice was weaker compared to that in *Gsk3b* ^f/f^ mice (**Figure** **4**A; Figure S4A, Supporting Information). The dynamic expression of ATGL suggested that it may play an important role in decidual cells lipolysis. To further confirm the relationship between ATGL and lipolysis in decidual cells, we treated both *Gsk3b* ^f/f^ mice and primary uterine cells with the ATGL inhibitor Atglistatin (ATGLi) on day 7. It was found that lipid accumulated in both cases (Figure 4B,C; Figure S4B,C, Supporting Information). These results indicated that *Gsk3b* may regulate lipid droplet degradation through ATGL.

To further investigate whether uterine *Gsk3b*‐regulated lipolysis in vivo depends on the kinase activity of GSK3β, we administered the GSK3 inhibitor LiCl to *Gsk3b* ^f/f^ mice via drinking water on day 7. LiCl treatment resulted in a similar accumulation of lipid droplets in day 8 uteri of *Gsk3b* ^f/f^ mice (Figure 4D; Figure S4D,E, Supporting Information). Given the central role of GSK3 in the Wnt signaling pathway, we hypothesized that GSK3β knockout animals might exhibit elevated canonical Wnt signaling in decidual cells. However, Western blotting revealed no significant difference in active β‐catenin levels, which represented the canonical Wnt activity, between the *Gsk3b* ^f/f^ and *Gsk3b* ^d/d^ mouse uteri (Figure 4E), and this may be attributed to compensation by GSK3α. We further investigated whether the canonical Wnt signaling pathway regulates lipolysis during decidualization. To this end, we treated mice with the Wnt pathway activator SKL2001, which does not act on GSK3β but inhibits the interaction between AXIN and β‐catenin,^[^ ^24^ ^]^ to increase nuclear localization of β‐catenin (Figure 4F,G). However, SKL2001 treatment did not suppress lipolysis (Figure 4H). These results suggest that GSK3β regulated lipolysis is independent of the canonical Wnt signaling during decidualization.

### 2.5 GSK3β Reduces the Protein Stability of RNF213

**Figure 5:**
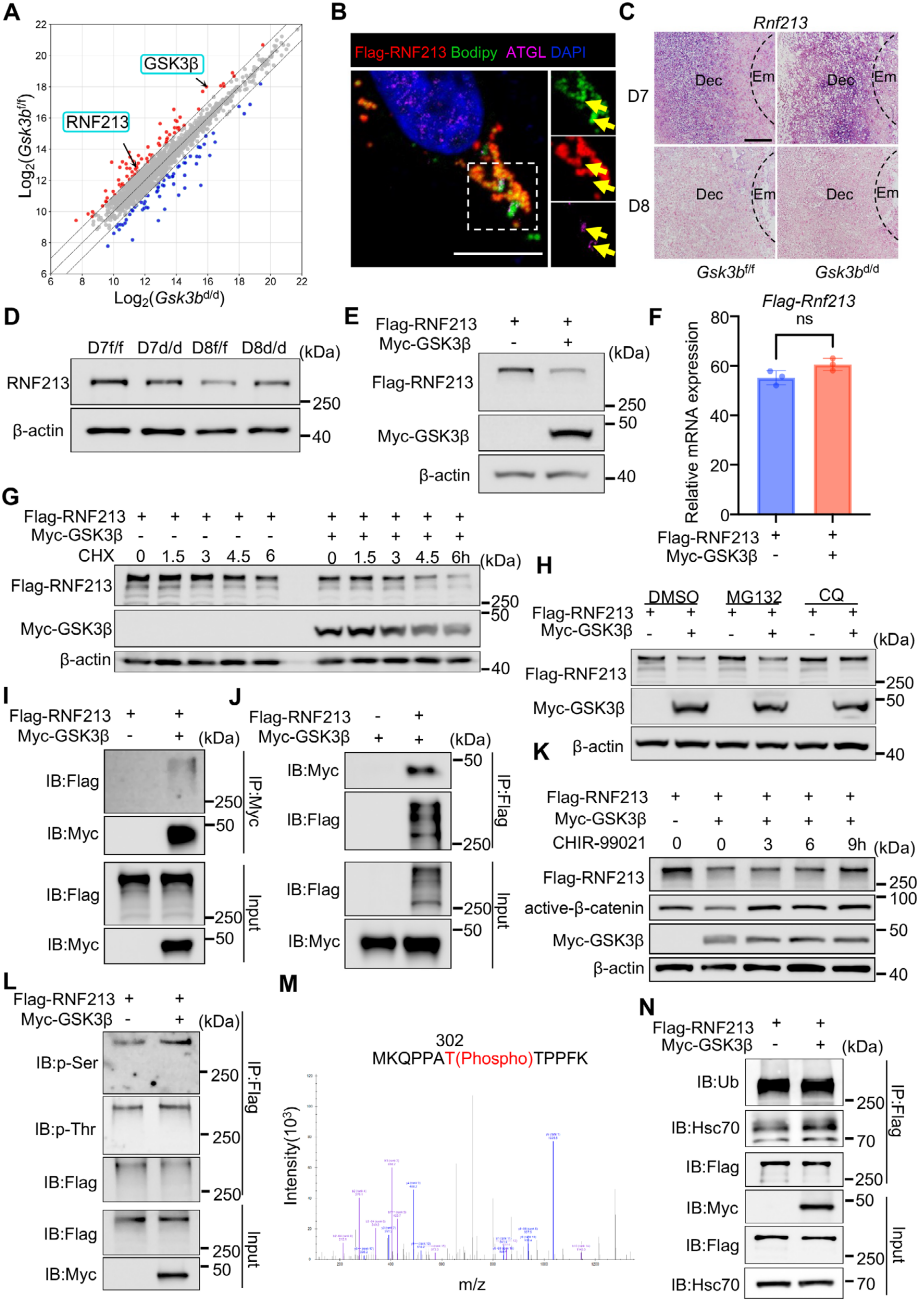
GSK3β phosphorylates RNF213, facilitating its degradation via the lysosomal pathway. A) Scatter plot analysis of the differential proteins interacting with GSK3β in *Gsk3b* ^f/f^ and *Gsk3b* ^d/d^ uteri on day 8 of pregnancy. B) Immunofluorescence analysis of ATGL, Bodipy, and overexpressed Flag‐RNF213 localization in HeLa cells. C) In situ hybridization of *Rnf213* in *Gsk3b* ^f/f^ and *Gsk3b* ^d/d^ uteri on day 7 and day 8. Scale bar: 200 µm. D) Western blot analysis of RNF213 in *Gsk3b* ^f/f^ and *Gsk3b* ^d/d^ implantation sites on day 7 and day 8. E) Western blot analysis revealed the protein level of Flag‐RNF213 with or without overexpression of Myc‐GSK3β in 293T cells. F) Quantitative real‐time PCR analysis of *Flag‐Rnf213* mRNA level with or without overexpression of Myc‐GSK3β in 293T cells (*N* = 3). The values are normalized to *Gapdh* and indicated as the mean ± SEM. Two‐tailed unpaired Student's t‐test. ns, not significant. G) Protein stability assay showing that the overexpression of Myc‐GSK3β markedly reduced the half‐life of Flag‐RNF213 proteins in 293T cells. 293T cells were treated with cycloheximide (50 µg mL^−1^) with or without overexpression of Myc‐GSK3β as indicated. H) Western blot analysis of Flag‐RNF213 with or without overexpression of Myc‐GSK3β in the presence of proteasome inhibitor MG132 (10 µm) or lysosomal inhibitor CQ (25 µm) in the indicated treatment group. I,J) 293T cells expressing Myc‐GSK3β and Flag‐RNF213. Immunoprecipitation with the indicated antibodies was performed. K) 293T cells were treated with GSK3β inhibitor CHIR‐99021 for the indicated times with expressing Myc‐GSK3β and Flag‐RNF213. L) Immunoprecipitation and Western blot assay to analyze the p‐Ser and p‐Thr in Flag‐RNF213. M) Mass spectrometry profile of Flag‐RNF213 peptide containing phosphorylated T302. N) The interaction between Flag‐RNF213 and Hsc70 was determined via Co‐IP with the indicated antibodies.

We next explored the molecular mechanism by which GSK3β may regulate lipolysis. We screened for proteins that interact with GSK3β and regulate lipolysis using mass spectrometry (MS), and identified RNF213 as a GSK3β‐interacting protein in day 8 uteri (**Figure** **5**A). RNF213 has been reported to compete with ATGL for position on lipid droplets, thereby inhibiting lipolysis.^[^ ^25^ ^]^ We first verified this report, and the results showed that ATGL could not localize on lipid droplets where RNF213 was present (Figure 5B). We next investigated the effect of GSK3β on RNF213, and observed that from day 7 to day 8, *Rnf213* mRNA expression was downregulated in the uteri of both *Gsk3b* ^f/f^ and *Gsk3b* ^d/d^ mice, whereas its protein level accumulated in *Gsk3b* ^d/d^ mice. (Figure 5C,D). We hypothesized that GSK3β may regulate lipolysis by modulating the protein level of RNF213. When Myc‐GSK3β and Flag‐RNF213 were overexpressed in 293T cells, it was found that overexpression of GSK3β reduced the protein levels of RNF213 in a dose‐dependent manner (Figure 5E; Figure S5A, Supporting Information). However, quantitative reverse transcription‐polymerase chain reaction (qRT‐PCR) analysis revealed no significantly alternation of *Rnf213* mRNA levels (Figure 5F). To determine whether the decreased level of RNF213 protein was due to decreased translational efficiency or increased degradation, the protein translation inhibitor cycloheximide (CHX) was used to measure the half‐life of RNF213 protein. Overexpression of GSK3β indeed accelerated the degradation of RNF213 (Figure 5G). These results indicate that GSK3β decreases the protein stability of RNF213.

### 2.6 GSK3β Promotes Lysosomal Degradation of RNF213 Through Phosphorylation at T302

To further investigate whether the lysosome or the proteasome was afforded for GSK3β‐accelerated RNF213 degradation, selective inhibitors of each process were used. The lysosomal inhibitor chloroquine (CQ) more effectively inhibited GSK3β overexpression‐induced RNF213 degradation compared to the proteasomal inhibitor MG132 (Figure 5H). Next, we used the exogenously expressed Myc‐GSK3β and Flag‐RNF213 to verify their physical interaction. Co‐IP experiments showed GSK3β interaction with RNF213 (Figure 5I,J). Since GSK3β is a protein kinase known to regulate protein degradation, and GSK3β‐regulated lipolysis depends on its kinase activity, we hypothesized that GSK3β may promote RNF213 turnover via phosphorylation. We observed that the GSK3 inhibitor CHIR‐99016 effectively inhibited RNF213 degradation, indicating that RNF213 degradation appears to be dependent on GSK3β‐regulated phosphorylation (Figure 5K). We next examined the effect of GSK3β on RNF213 phosphorylation status using pan‐serine or threonine phosphorylation‐specific antibodies. The results showed that GSK3β overexpression increased the phosphorylation of serine and threonine residues on RNF213 (Figure 5L). To identify the potential GSK3β phosphorylation site(s) on RNF213, we overexpressed Flag‐RNF213 with or without Myc‐GSK3β in 293T cells, then isolated Flag‐RNF213 by immunoaffinity purification and identified phosphorylated sites by liquid chromatography‐tandem mass spectrometry (LC‐MS/MS) (Figure S5B, Supporting Information). We identified two differential phosphorylation sites, T302 and S1258, when Myc‐GSK3β and Flag‐RNF213 were co‐expressed (Figure 5M; Figure S5C, Supporting Information). However, only the T302 residue is evolutionarily conserved and was chosen for the following study (Figure S5D, Supporting Information). To explore the lysosomal degradation mechanism of phosphorylated RNF213, we analyzed its amino acid sequence and identified multiple classical Hsc70 binding motifs (Table S2, Supporting Information), indicating that RNF213 might be degraded in the lysosome via chaperone‐mediated autophagy (CMA) pathway.^[^ ^26^ ^]^ Indeed, it was found that RNF213 can bind to Hsc70, and overexpression of GSK3β increased binding of RNF213 to Hsc70 (Figure 5N), supporting the idea that phosphorylated RNF213 is degraded through CMA.

### 2.7 *Gsk3b* Deficiency Primarily Affects Lipid Composition in Decidual Cells

**Figure 6:**
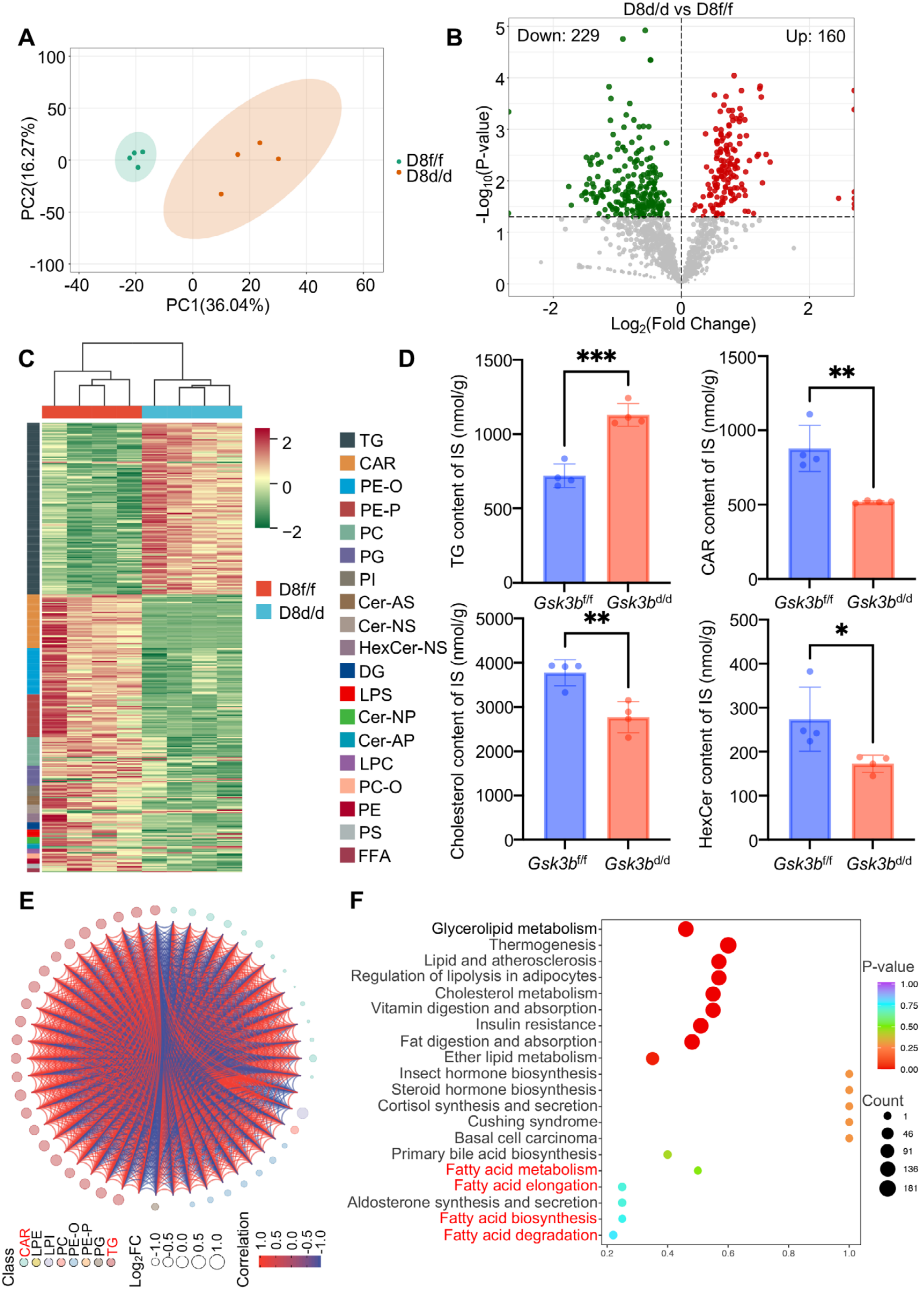
*Gsk3b* ^d/d^ decidual cells display significant alterations of lipid composition. A) PCA plot of the lipidomics profiling data showing clustering of *Gsk3b* ^f/f^ and *Gsk3b* ^d/d^ decidual tissues on day 8. B) Volcano plot showing the significantly changed lipid species between *Gsk3b* ^f/f^ and *Gsk3b* ^d/d^ decidual tissues on day 8. C) Heatmap showing the relative abundance of lipids between *Gsk3b* ^f/f^ and *Gsk3b* ^d/d^ decidual tissues on day 8. D) Bar graphs showing the levels TGs, CAR, Cholesterol, and HexCer in *Gsk3b* ^f/f^ (*N* = 4 animals) and *Gsk3b* ^d/d^ (*N* = 4 animals) decidual tissues on day 8. Data represent the mean ± SEM. Two‐tailed unpaired Student's *t*‐test, ^***^ *p* = 0.0003, ^**^ *p* = 0.0036 (CAR), ^**^ *p* = 0.0048 (Cholesterol), ^*^ *p* = 0.0354. E) Chord diagram delineates intergroup correlation patterns among the top 50 differentially expressed lipids. F) KEGG pathway analysis for differentially expressed lipids between *Gsk3b* ^f/f^ and *Gsk3b* ^d/d^ decidual tissues on day 8.

Having established the role of *Gsk3b* in regulating lipid droplets, we next sought to investigate the biological functions of this lipid degradation in the mouse uterus. Decidual tissues collected from *Gsk3b* ^f/f^ and *Gsk3b* ^d/d^ uteri on days 7 and 8 were subjected to metabolomics analysis. PCA analysis revealed distinct clusters for *Gsk3b* ^f/f^ and *Gsk3b* ^d/d^ samples (Figure S6A, Supporting Information). The heatmap and volcano plot indicated a widespread variation in metabolites in the uteri of *Gsk3b* ^f/f^ mice from day 7 to day 8. However, the differential metabolites between *Gsk3b* ^f/f^ and *Gsk3b* ^d/d^ mice on day 8 are concentrated in specific components (Figure S6B,C, Supporting Information). The heatmap of differential metabolites on day 8 indicated that *Gsk3b* knockout results in significant differences in fatty acid metabolites, whereas other metabolites remain largely unchanged (Figure S6D, Supporting Information). To gain further insights into lipid metabolites, we performed quantitative lipidomics in day 8 decidual tissues of *Gsk3b* ^f/f^ and *Gsk3b* ^d/d^ mice. PCA analysis suggested a significantly different lipid metabolite status between *Gsk3b* ^f/f^ and *Gsk3b* ^d/d^ mice (**Figure** **6**A). A total of 389 lipid compounds exhibited differential levels in *Gsk3b* ^d/d^ mice compared to wild type, with 229 downregulated and 160 upregulated (Figure 6B). The lipidomics profile revealed a notable increase in triglycerides (TG) in *Gsk3b* ^d/d^ mice. In contrast, levels of other lipid compounds, including acylcarnitines (CAR), cholesterol, glycosphingolipids (HexCer), diglycerides (DG), and ether‐linked phospholipids, were generally lower, with the most significant reduction observed in CAR (Figure 6C,D; Figure S6E–G, Supporting Information). Once long‐chain fatty acids are activated to form acyl‐CoA, they are transported into the mitochondria as CAR, facilitated by CPT1. In the mitochondria, CAR are converted back to acyl‐CoA by CPT2, allowing acyl‐CoA to proceed with β‐oxidation, ultimately producing acetyl‐CoA. The correlation and chord diagram of the top fifty differential metabolites revealed a significant negative correlation between TG and CAR (Figure 6E). Enrichment analysis of these metabolites identified pathways associated with fatty acid metabolism (Figure 6F). Furthermore, electron microscopy images of decidual cells showed contacts between the lipid droplets and the mitochondria (Figure 2F). Therefore, the observed decrease in CAR levels in *Gsk3b* ^d/d^ mice may be due to reduced availability of fatty acids from TG hydrolysis.

### 2.8 Defective Lipolysis Results in Decreased Acetyl‐CoA Levels and Reduced Histone Acetylation in *Gsk3b* Knockout Mice

**Figure 7:**
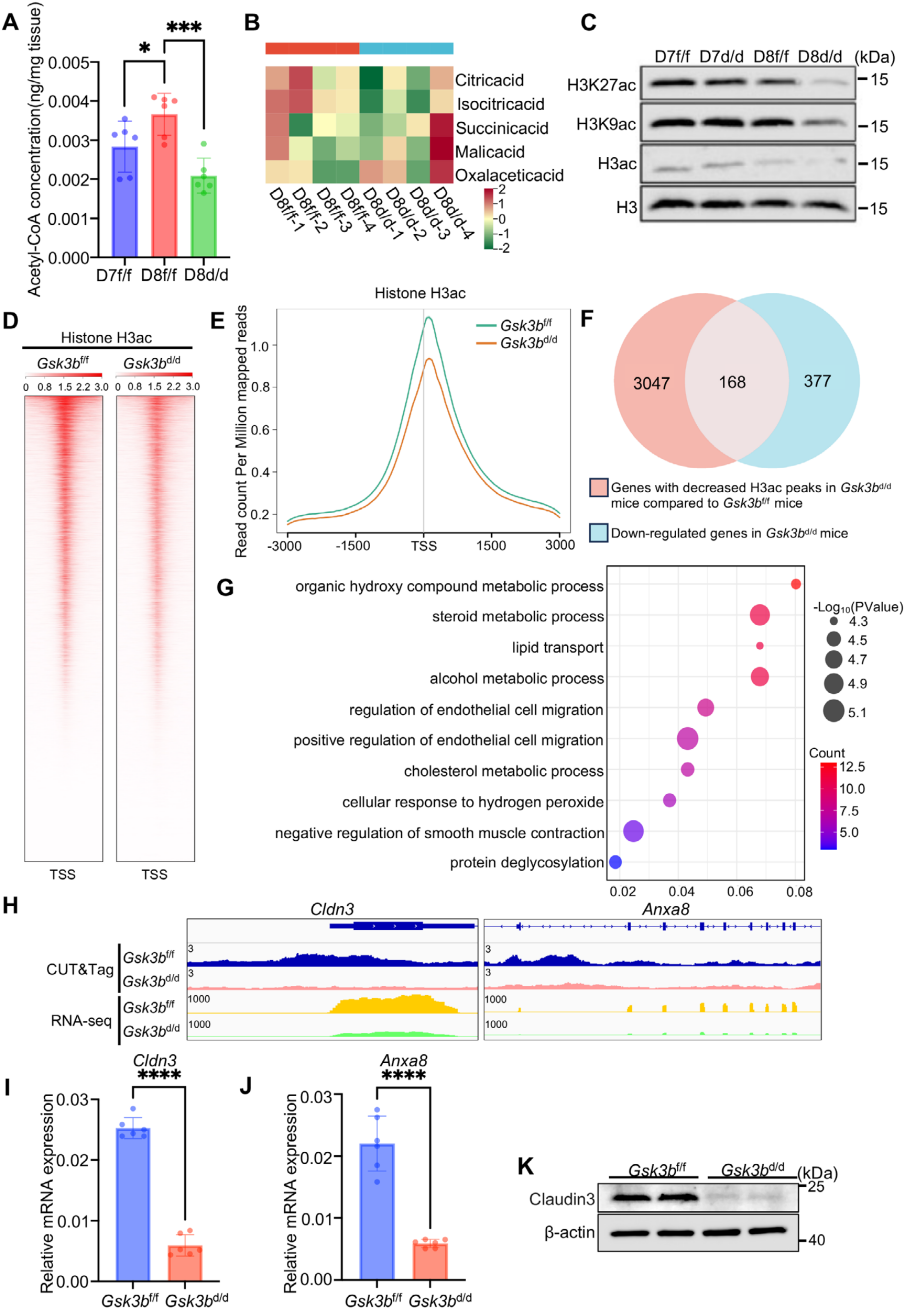
Defective lipolysis in the absence of *Gsk3b* results in decreased acetyl‐CoA levels and reduced histone acetylation in decidual cells. A) Acetyl‐CoA content assay of *Gsk3b* ^f/f^ (*N* = 3 animals) decidual tissues on day 7 and day 8 as well as *Gsk3b* ^d/d^ (n = 3 animals) decidual tissues on day 8. Data represent the mean ± SEM. Two‐tailed unpaired Student's *t*‐test, ^*^ *p* = 0.0371, ^***^ *p* = 0.0003. B) Heatmap showing the relative abundance of metabolites associated with the tricarboxylic acid cycle between *Gsk3b* ^f/f^ and *Gsk3b* ^d/d^ decidual tissues on day 8. C) Western blot analysis of H3K27ac, H3K9ac, and H3ac in *Gsk3b* ^f/f^ and *Gsk3b* ^d/d^ implantation sites on day 7 and day 8. D) Coverage profiles for histone H3ac. Heatmap of peaks with decreased histone H3ac following *Gsk3b* deletion centered at TSS. TSS, transcriptional start site. E) Profile plot showing the distribution of normalized H3ac CUT&Tag signals. TSS, transcriptional start site. F) Venn diagram showing the overlap between genes with decreased H3ac peaks by *Gsk3b* loss (*N* = 3215) and down‐regulated genes upon *Gsk3b* loss (*N* = 545). G) Gene Ontology functional analysis for 168 overlapped genes using DAVID by Kappa Statistics (*p* < 0.05). H) Genome browser view of normalized H3ac CUT&Tag signals and RNA‐seq tracks for representative genes, *Cldn3* and *Anxa8*. I‐J) Quantitative real‐time PCR analysis of *Cldn3* and *Anxa8* mRNA levels in *Gsk3b* ^f/f^ (*N* = 3 animals) and *Gsk3b* ^d/d^ (*N* = 3 animals) implantation sites on day 8. The values are normalized to *Gapdh* and indicated as the mean ± SEM. Two‐tailed unpaired Student's *t*‐test, ^****^ *p* < 0.0001. K) Western blot analysis of Claudin3 in *Gsk3b* ^f/f^ and *Gsk3b* ^d/d^ implantation sites on day 8.

CAR is converted into acyl‐CoA in the mitochondria, where it plays a key role in β‐oxidation to produce acetyl‐CoA. We then measured the acetyl‐CoA levels in decidual tissue. The results showed that acetyl‐CoA levels increased from day 7 to day 8 in *Gsk3b* ^f/f^ mice, whereas the acetyl‐CoA content in *Gsk3b* ^d/d^ mice was lower than that in *Gsk3b* ^f/f^ mice on day 8 (**Figure** **7**A). Acetyl‐CoA in the mitochondria can act as a substrate for the tricarboxylic acid (TCA) cycle. However, the results of the metabolomic analysis indicated no significant differences in detectable TCA cycle metabolites between *Gsk3b* ^f/f^ and *Gsk3b* ^d/d^ mouse decidual tissues on day 8 (Figure 7B). Besides its role in energy production, acetyl‐CoA is also the substrate for histone acetylation.^[^ ^27^ ^]^ We next explored whether the decreased levels of acetyl‐CoA in *Gsk3b* ^d/d^ mice could impact histone acetylation, which is linked to chromatin opening and transcription. By using antibodies targeting pan‐acetylation of H3, including acetylation at H3K9, H3K14, H3K18, H3K23, and H3K27, along with antibodies specific for acetylated H3K9 and H3K27 sites, we found that the *Gsk3b* ^d/d^ decidual tissues showed reduced levels of pan‐histone H3, H3K9, and H3K27 acetylation (Figure 7C). To precisely map the altered histone acetylation sites in the genome, we performed a Cleavage Under Targets and Tagmentation (CUT&Tag) experiment on both *Gsk3b* ^f/f^ and *Gsk3b* ^d/d^ day 8 decidual cells, utilizing antibodies that target pan‐acetylated H3, acetylated H3K9, and H3K27. In line with the findings from the immunoblot assay, the decidual cells from *Gsk3b* ^d/d^ mice exhibited lower peaks of histone H3 acetylation (Figure 7D,E; Figure S7A–C, Supporting Information). Next, to identify the specific genes influenced by histone H3 acetylation that contribute to the abnormal decidualization, we conducted an integrated analysis. This involved comparing genes associated with reduced histone H3 acetylation peaks, with transcriptionally downregulated genes in the decidual cells of *Gsk3b* ^d/d^ mice versus *Gsk3b* ^f/f^ mice. We identified 168 target genes potentially regulated by histone H3 acetylation (Figure 7F). We also performed pathway enrichment analysis for these different expressed genes and identified enrichment in pathways such as endothelial cell migration and steroid metabolic process (Figure 7G *)*. It has been previously confirmed that knockout of *Cldn3* in female mice results in impaired fertility, decreased expression of genes related to decidualization.^[^ ^28^ ^]^ Knockdown of *Anxa8* expression in mice reduces the number of embryo implantation sites.^[^ ^29^ ^]^ Notably, the peaks of histone H3 acetylation were significantly reduced at the loci of both *Cldn3* and *Anxa8* (Figure 7H; Figure S7D, Supporting Information). Consistently, *Gsk3b* loss decreased *Cldn3* and *Anxa8* expression in decidual cells on day 8 of pregnancy (Figure 7I–K), highlighting the critical role of histone acetylation mediated induction of target genes, such as *Cldn3* and *Anxa8* in decidualization processes.

In summary, our study uncovered the regulatory mechanism and function of timely lipolysis during decidualization. We provide genetic evidence for an essential role of GSK3β by inducing the degradation of RNF213 in this process. We found that the T302 site of RNF213 can be phosphorylated by GSK3β, which leads to lysosomal degradation of RNF213, ensuring the localization of ATGL on lipid droplets to facilitate lipid droplet degradation. This process releases free fatty acids, which can then enter the mitochondria for β‐oxidation to produce acetyl‐CoA. Acetyl‐CoA plays a pivotal role in cellular metabolism and can influence histone acetylation modifications, influencing the expression of decidualization‐related genes. The ablation of *Gsk3b* disrupted the downregulation of RNF213, hindering the rapid breakdown of lipid droplets and leading to a diminished supply of acetyl‐CoA. Consequently, the resulting decrease in histone acetylation modification was inadequate to support expression of genes associated with decidualization.

## 3 Discussion

In this study, we demonstrate that uterine GSK3β is a critical regulator of histone acetylation via regulating levels of acetyl‐CoA through its role in lipolysis, facilitating terminal differentiation of decidual cells essential for normal embryonic development.

During decidualization progression, we observed that decidual cells accumulate lipid droplets, which peak on day 7 of pregnancy. Our previous studies have shown that primary trophoblast giant cells secrete LPL to facilitate lipid accumulation in the decidua, suggesting the role of embryonic signals in decidualization.^[^ ^12^ ^]^ Furthermore, it was noticed that the lipid droplet undergone rapid lipolysis from day 7 to day 8, and the significant upregulated CAR in decidual cells strongly suggested the usage of fatty acid for β‐oxidation by the decidual cells during the terminal differentiation in vivo. Decidualization is likely an energy‐intensive process, with numerous studies demonstrating the significance of glucose metabolism in preparing the endometrium and facilitating decidualization.^[^ ^5^, ^30^, ^31^ ^]^ Beyond glucose, various cell types utilize lipids as an alternative energy source. Fatty acids, derived from triglycerides, yield more ATP per gram compared to carbohydrates or proteins. In this study, we observed the metabolism transition to lipid mobilization and β‐oxidation when the anti‐mesometrial decidual cells progress to terminal differentiation on day 8. It was recently reported that the developing embryo requires plenty of glucose in this stage,^[^ ^31^ ^]^ whether the metabolism transition of the decidual cell would facilitate more glucose transport into the embryo needs further exploration.

The β‐oxidation pathway is a well‐conserved evolutionary process that metabolizes fatty acids within the mitochondria to generate acetyl‐CoA, a key molecule involved in histone acetylation and non‐histone modifications. The uterine cell differentiation was mainly regulated at the transcriptional level by estrogen and progesterone, and the epigenetic regulation, such as various histone modifications, has been reported to attend these processes. Our recent research has revealed that *Men1* regulates PTX3 expression by modulating H3K4me3 modifications, which subsequently impacts the balance of BMP2 and FGF2 during decidualization.^[^ ^32^ ^]^ Additionally, the loss of EZH2 results in the formation of ectopic myofibroblasts and collagen deposition in the decidua through the TGF‐β signaling pathway.^[^ ^33^ ^]^ Estradiol treatment has been shown to increase histone acetylation modifications in the uteri of both mice and rats.^[^ ^34^, ^35^ ^]^ Additionally, blockage of histone deacetylase by HDAC inhibitors enhances the proliferative effects of estradiol on the mouse uterus.^[^ ^36^, ^37^ ^]^ In human endometrial stromal cells, decidualization results in a genome‐wide increase in the levels of acetylation of histone H3 at lysine 27 (H3K27ac).^[^ ^37^ ^]^ These findings indicate that histone acetylation is essential for regulating uterine receptivity and facilitating decidualization during the implantation process. In this study, we found that in wild‐type mice, the overall level of histone acetylation in decidual cells declines from day 7 to day 8 of pregnancy. Nevertheless, the processes of adding and removing histone acetylation maintain a dynamic equilibrium. This balance is disturbed in the absence of *Gsk3b*, resulting in decreased histone acetylation levels at particular genomic regions. Our data are consistent with β‐oxidation produced acetyl‐CoA being critical for the histone acetylation when decidual cells undergo terminal differentiation.

The decidualization process also involves significant lipid remodeling. During days 4–8 of pregnancy in mice, decidual cells accumulate phospholipids, accompanied by significant alterations in their distribution across various cellular regions at the implantation site.^[^ ^7^, ^38^ ^]^ The de novo synthesis of sphingolipids is essential for implantation and plays a crucial role in the decidualization process in mice.^[^ ^11^ ^]^ Additionally, fatty acids generated from lipolysis are vital for the synthesis of functional lipids and may influence decidualization by modulating lipid remodeling. The process of lipid droplet breakdown that ultimately generates acetyl‐CoA inevitably involves energy production and lipid remodeling, both of which may significantly influence the decidualization process. However, we have not delved deeply into these aspects. Additionally, previous reports have indicated that, on day 8 of pregnancy, long‐chain fatty acid transport protein 4 (FATP4) is expressed in the yolk sac and trophoblast cells surrounding the yolk sac cavity, suggesting that FATP4 may play a critical role in fat absorption during early embryonic development.^[^ ^39^ ^]^ Whether decidual cells, as fatty acid‐producing cells, participate in lipid crosstalk with the embryo requires further investigation.

GSK3β is a classical protein kinase that regulates numerous fundamental biological processes through the phosphorylation of serine and threonine residues.^[^ ^14^ ^]^ Research of GSK3β functions in lipid catabolism predominantly emphasizes its inhibitory effects. Studies have demonstrated that GSK3β suppresses expression of lipolysis‐promoting genes, such as *Pnpla2* (which encodes the ATGL) via PPARα signaling.^[^ ^40^ ^]^ However, there are limited reports regarding the promotional effects of GSK3β on lipolysis. In this study, we observed that GSK3β facilitated the degradation of lipid droplets during the process of decidualization, and thus influenced the lipid profile of decidual cells, suggesting a cellular‐context‐specific regulation of GSK3β on lipid metabolism.

Among the proteins that interact with GSK3β, RNF213 has been implicated in the regulation of lipolysis. RNF213, characterized by its ubiquitin ligase activity and putative mechanical ATPase functions, contains a RING finger domain along with two adjacent AAA+ modules. Recent studies have shown that RNF213 localizes to lipid droplets, where it competes with ATGL, thus inhibiting lipolysis.^[^ ^25^ ^]^ Our investigation highlighted the critical role of ATGL in lipolysis during decidualization. On day 8 of pregnancy in GSK3β‐deficient mice, the RNF213 content in the uterus was higher than that in wild‐type mice, which may lead to the inhibition of ATGL localization on lipid droplets. In vitro experiments further showed that GSK3β‐mediated phosphorylation of RNF213 enhances its degradation via lysosomal pathways. In our experiments, we also noticed a decrease in RNF213 mRNA expression in decidual cells on the antimesometrial pole from day 7 to day 8 of pregnancy. This suggested that RNF213 is regulated not only at the protein level by GSK3β, but that reduced transcriptional expression may be required to promote rapid degradation of lipid droplets.

As the rate‐limiting enzyme for triglyceride hydrolysis, ATGL has been extensively studied regarding its regulation at both the transcriptional and protein levels. Evidence indicates that *Pnpla2* is a direct target of the PPAR family of nuclear receptor transcription factors,^[^ ^41^ ^]^ while phospho‐STAT5 directly activates *Pnpla2* transcription in white and brown adipocytes.^[^ ^42^, ^43^ ^]^ At the protein level, ATGL is regulated by phosphorylation and palmitoylation, which affect its enzymatic activity and localization.^[^ ^44^, ^45^, ^46^, ^47^ ^]^ In our experiments, we primarily focused on how GSK3β influences ATGL localization on lipid droplets via RNF213 to inhibit lipolysis. However, RNA‐seq results revealed that *Gsk3b* knockout also resulted in a reduction of *Pnpla2* transcription levels, suggesting that GSK3β may regulate lipolysis through ATGL at the transcriptional level as well. A detailed analysis of these regulatory mechanisms warrants further investigation.

In conclusion, our experiments identify a critical role of GSK3β in the decidualization process in mice, providing new insights into the function of lipid metabolism and its molecular regulatory network during decidualization. Our findings will significantly advance our understanding of the current model of decidualization and inspire future research aimed at improving human fertility.

## 4 Experimental Section

### Animals and Treatments

*Gsk3b* ^f/f^ mice were generated as previously described.^[^ ^48^ ^]^ Uterine‐specific knockout mice were created by crossing *Gsk3b* ^f/f^ mice with Pgr‐Cre mice. All mice were housed in the animal care facility at Xiamen University under controlled conditions (22 ± 2 °C, 50–60% humidity, 12‐h light/dark cycle, lights on at 7 AM) with free access to food and water, following the institutional guidelines for laboratory animal care and use. Female mice were mated with fertile wild‐type male mice to induce pregnancy (vaginal plug = day 1 of pregnancy). Plug‐positive females were kept separately for pregnancy experiments. On days 5 and 6 of pregnancy, implantation sites were identified through an intravenous injection of 100 µL of 1% Chicago blue dye in saline. Distinct blue bands marking implantation sites were recorded, and the number and average weight of the implantation sites were noted. Blood samples were collected from pregnant mice on day 8, and serum levels of estradiol (E2) and progesterone (P4) were measured using radioimmunoassay. All experimental procedures were approved by the Animal Welfare Committee of Research Organization (XMULAC20210105), Xiamen University.

### In Situ Hybridization

In situ hybridization (ISH) was conducted following previously described protocols.^[^ ^32^ ^]^ Briefly, fresh frozen tissue sections (10 µm) were mounted onto poly‐L‐lysine‐coated slides and fixed in 4% paraformaldehyde at room temperature for 1 h. Following prehybridization, the sections were hybridized with specific cRNA probes and incubated overnight at 55 °C. The next day, the sections were treated with RNase A (10 mg mL^−1^) at 37 °C for 30 min. RNase A‐resistant hybrids were detected using NBT/BCIP (Beyotime Biotechnology, C3206).

### Immunostaining

Paraffin Sections (5 µm) were utilized for immunohistochemistry (IHC), Tissue specimens were fixed overnight in 10% neutral buffered formalin, followed by dehydration in increasing concentrations of ethanol and clearing with xylene. Uterine slices were deparaffinized and incubated in citrate buffer for antigen retrieval using hyperbaric heating. The slices were then incubated overnight at 4 °C with primary antibodies. Antigens were visualized using a Histostain‐SP Kit (Zhongshan Golden Bridge Biotechnology).

Frozen sections (10 µm) and cell culture climbing slides were used for immunofluorescence (IF) staining. Slices were fixed in 4% paraformaldehyde for 15 min and permeabilized with 0.1% Triton X‐100 at room temperature for 15 min. The remaining steps followed the standard IHC protocol, with fluorophore‐labeled secondary antibodies (Jackson ImmunoResearch, 1:200) used for antigen detection. Nuclei were stained with 4,6‐diamidino‐2‐phenylindole (DAPI, 1 µg mL^−1^, Solarbio). Imaging was conducted using either a Nikon (Ni‐U) fluorescence microscope or a Zeiss LSM 880+Airyscan. Detailed information on the antibodies used has been summarized in the Supporting Information (Table S3, Supporting Information).

### Nuclear and Cytoplasmic Fractionation

Fresh decidual tissue samples were minced into small pieces and homogenized in tissue lysis buffer prepared by mixing extraction reagents A and B (20:1, v/v) with 1 mM PMSF on ice. Tissue was homogenized at a ratio of 60 mg tissue per 200 µL lysis buffer (≤30 mg tissue per 100 µL). Homogenates were incubated on ice for 15 min and centrifuged at 1500 × g for 5 min at 4 °C. The supernatant was collected as the cytoplasmic fraction. The pellet was further processed following the manufacturer's instructions to extract nuclear and remaining cytoplasmic proteins.

### Western blotting

Protein extraction and Western blot analysis were conducted following established protocols.^[^ ^49^ ^]^ Briefly, proteins were separated by 10% SDS‐PAGE, blotted onto methanol‐activated polyvinylidene difluoride (PVDF) membrane, and incubated overnight at 4 °C with primary antibodies. After incubation of secondary antibodies (Zhongshan Golden Bridge Biotechnology, 1:5000), film exposure was performed and visualized by E‐Blot Touch Imager (Shanghai e‐Blot Photoelectric Technology Co., Ltd). β‐actin was used as a loading control, while H3 served as a nuclear protein control. Detailed information on the antibodies used has been summarized in the Supporting Information (Table S3, Supporting Information).

### Inhibitor Treatment

For cell experiments, the inhibitor was dissolved in DMSO to obtain a stock solution and subsequently diluted with culture medium to the desired working concentration. The final concentration of DMSO was adjusted to 0.1% in both inhibitor‐treated and vehicle control groups.

For animal experiments, inhibitors were first dissolved in DMSO to prepare stock solutions and further diluted with the vehicle solution before administration. The final concentration of DMSO for intraperitoneal injection did not exceed 10%, and vehicle control mice received injections containing the same concentration of DMSO without inhibitors. The ATGL inhibitor Atglistatin (ATGLi) and the Wnt pathway activator SKL2001 were administered via intraperitoneal injection twice on day 7 of pregnancy (10:00 a.m. and 10:00 p.m.). LiCl (lithium chloride) was administered orally via drinking water on day 7. The inhibitor was dissolved in the drinking water, and the dosage was adjusted according to an estimated daily water consumption of 5 mL per mouse. Detailed information on the inhibitors in this study was provided in Table S4 (Supporting Information).

### Quantitative Real‐Time PCR

Total RNA was isolated from uterine tissues or cells using TRIzol reagent (Invitrogen) according to the manufacturer's instructions. Approximately 1 µg of RNA was used for cDNA synthesis. Gene expression levels were assessed through quantitative real‐time PCR (qRT‐PCR) using SYBR Green (TAKARA). All assays were performed at least three times.

### Immunoprecipitation and Immunoblotting Analysis

Proteins were extracted from cultured cells or uterine tissues using lysis buffer containing 50 mM Tris (pH 7.4), 150 mM NaCl, 1% Nonidet P‐40, 0.5% sodium deoxycholate, and 0.1% SDS. The cell extracts were clarified by centrifugation at 12000 × g, and the resulting supernatants (2 mg protein/mL) were used for immunoprecipitation with the specified antibodies. After overnight incubation at 4 °C, recombinant Protein A/G magnetic beads, which combine IgG‐binding domains of Protein A and Protein G, were added and incubated for 3 additional h. Immunocomplexes were washed three times with lysis buffer and analyzed by immunoblotting using the corresponding antibodies.

### Mass Spectrometry Analysis

Protein samples were separated in SDS‐PAGE and visualized with Coomassie blue stain. After staining of gels with Coomassie blue, excised gel segments were subjected to in‐gel trypsin digestion and dried. Samples were analyzed on a nanoElute (Bruker) coupled to a timsTOF Pro (Bruker) equipped with a CaptiveSpray source. Peptides were dissolved in 10 µL 0.1% formic acid and were auto‐sampled directly onto a homemade C18 column (35 cm × 75 µm i.d., 1.9 µm 100Å). Samples were then eluted for 60 min with linear gradients of 3–35% acetonitrile in 0.1% formic acid at a flow rate of 300 nL/min. Mass spectra data were acquired with a timsTOF Pro mass spectrometer (Bruker) operated in PASEF mode. The raw files were analyzed by Peaks Studio X software against the Uniprot database. Precursor and fragment mass tolerances were set to 20 ppm and 0.05 Da, and semispecific Digest mode was allowed. Up to three missed cleavages per peptide were allowed. Oxidation of methionine and acetylation of protein N‐termini were set as variable modifications. Phosphorylation of Serine, Threonine, and Tyrosine was set as variable modifications for phosphoproteomics. Carbamidomethylation of Cysteine was set as a fixed modification.

### Primary Uterine Stromal Cell Culture

Decidua from day 7 pregnant wild‐type C57BL/6 mice (embryo and muscle layer removed) was dissected and chopped into small pieces. The tissue fragments were initially digested in fresh HBSS medium (Gibco) containing 6 mg/mL dispase (Gibco) and 25 mg mL^−1^ pancreatin (Sigma), followed by incubation in medium with 0.5 mg mL^−1^ collagenase (Sigma) at 37 °C for 30 min. Digested cells were filtered through a 70 µm sieve to isolate stromal cells. The stromal cells were cultured in 60‐mm dishes or 6‐well plates containing phenol red‐free Dulbecco's Modified Eagle Medium (DMEM) and Ham's F12 nutrient mixture (1:1) (Gibco) supplemented with 10% charcoal‐stripped fetal bovine serum (CS‐FBS), 10 nM E2 (Sigma), 1 µm P4 (Sigma), and antibiotics. After 2 h, the medium was refreshed with new medium of the same composition.

### Detection of Lipid Droplets with Lipophilic Dyes

BODIPY 493/503 (MCE, HY‐W090090) and Oil Red O are commonly used lipophilic dyes for staining. For BODIPY 493/503 staining, frozen sections (10 µm) were fixed in 4% paraformaldehyde for 10 min. The sections were stained with BODIPY 493/503 (MCE, HY‐W090090, 1:1500) for 10 min at room temperature, washed three times with PBS, and counterstained with 1 mg mL^−1^ 4,6‐diamidino‐2‐phenylindole (DAPI; Beyotime, C1002) diluted to 1:1000 in PBS for 15 min at room temperature. For Oil Red O staining, the dye was mixed with ddH2O in a 3:2 ratio, filtered, and incubated at room temperature for 10 min. The mixture was then applied to frozen sections pre‐soaked in a 60% isopropanol solution. After staining, the sections were washed with PBS and mounted with glycerin gelatin.

### Measurement of TG and Acetyl‐CoA Level in Uterine Tissues

The Triglyceride Content Determination Kit (Solarbio, BC0620) and the Acetyl‐CoA ELISA Kit (Elabscience, E‐EL‐0125) were used to measure total triglyceride and acetyl‐CoA levels in uterine tissues. Following the removal of myometrium and embryonic tissues from the uteri, the decidua was weighed and then homogenized at 4 °C. The supernatant obtained after refrigerated centrifugation was processed according to the manufacturer's protocol. Optical density (OD) values were measured spectrophotometrically using a SpectraMax 190 Microplate Reader (Molecular Devices), and the absolute amounts of triglycerides and acetyl‐CoA were normalized to the weight of the decidual tissue. Each group included at least three biological replicates, and each assay was performed with at least two technical replicates.

### CUT&Tag

The Cleavage Under Targets and Tagmentation (CUT&Tag) procedure was performed using the NovoNGS CUT&Tag 4.0 High‐Sensitivity Kit (Novoprotein, N259‐YH01). In brief, each sample, containing 100000 decidual cells, was first incubated with ConA magnetic beads for 10 min at room temperature. Following this, the primary antibody was applied overnight at 4 °C. The next day, secondary antibody incubation was performed for 1 h at room temperature, followed by ChiTag transposome treatment for an additional hour, and Tn5 tagmentation at 37 °C for 1 h. DNA was then extracted, amplified, and purified. The sequencing was performed using the Illumina NovaSeq 6000 platform.

### Metabolomics and Lipidomics

Sampels were removed from storage at −80 °C and placed on ice to thaw. 50 µL of pure methanol was added, mixed thoroughly using a vortex, and centrifuged at 12 000 rpm at 4 °C for 30 s. Samples were homogenized with a steel ball at 30 Hz for 20 s, followed by another centrifugation at 3,000 rpm at 4 °C for 30 s. 1 mL of extraction solvent (MTBE: MeOH = 3:1, v/v) containing an internal standard mixture was then added, and the solution vortexed for 15 min, followed by the addition of 200 µL of ultrapure water. After vortexing again for 1 minute and centrifugation at 12 000 rpm for 10 min, 200 µL of the upper organic phase was carefully withdrawn and evaporated using a vacuum concentrator. The dried residue was resuspended in 200 µL of reconstitution solution (ACN: IPA = 1:1, v/v) for subsequent LC‐MS/MS analysis.

### Statistical Analysis

Statistical analyses were conducted in GraphPad Prism 9 software. The data were expressed as the mean ± standard deviation (SD) unless otherwise specified. Two‐tailed Student's *t*‐test was used to compare the two groups. The signal intensity for Western blot and Immunohistochemistry was carried out using the ImageJ software. The level of each band for Western blot was normalized to the level of β‐actin. The signal intensity for Immunohistochemistry was normalized to the mean of the control group. The sample size (n) was shown in the corresponding Figure legends. Statistical significance was defined as: ^*^ *p* < 0.05; ^**^ *p* < 0.01; ^***^ *p* < 0.001; ^****^ *p* < 0.0001;ns, not significant.

## Author Contributions

P.W. and Y.T. contributed equally to this work. P.W., Y.T., X.Z., H.Z., E.Z., G.L., H.C., Y.W. performed experiments. W.D. and Y.N. conducted the bioinformation analysis. J.W. provided the Gsk3b mouse model and edited the manuscript. P.W., S.K., H.B., H.W., and Z.L. analyzed data. H.W., S.K., H.B. and Z.L. designed the studies. P.W., S.K. and H.B. wrote the manuscript.

## Conflict of Interest

The authors declare no competing interests.

## Supporting information



Supporting Information

Supporting Data File

## Data Availability

The RNA‐seq and ChIP‐seq data generated in this study have been deposited in the National Center for Biotechnology Information Sequence Read Archive under accession number PRJNA1242492.
