# Supplementary material for: GSK3β‐Regulated Lipolysis is Required for Histone Acetylation and Decidualization in Early Pregnancy
Source: Adv Sci (Weinh). 2025 Nov 9;13(5):e14291. doi: 10.1002/advs.202514291 (PMC12850330; doi:10.1002/advs.202514291)
Supplement: Supplementary file 1 — Supporting Information [file ADVS-13-e14291-s002.docx]

**GSK3β-Regulated Lipolysis Is Required for Histone Acetylation and Decidualization in Early Pregnancy**

Peiran Wang^1, 2, #^, Yedong Tang^2, #^, Xueling Zhao^2^, Yu Ni^2^, Hualan Zhou^2^, Enhao Zhang^1, 2^, Gaizhen Li^2^, Han Cai^2^, Yinan Wang^2^, James R. Woodgett^3^, Wenbo Deng^2^, Haibin Wang^2, 4, *^, Zhongxian Lu^1, 5, *^, Haili Bao^2, *^, Shuangbo Kong^2,^ ^*^

**Supplementary materials**

**
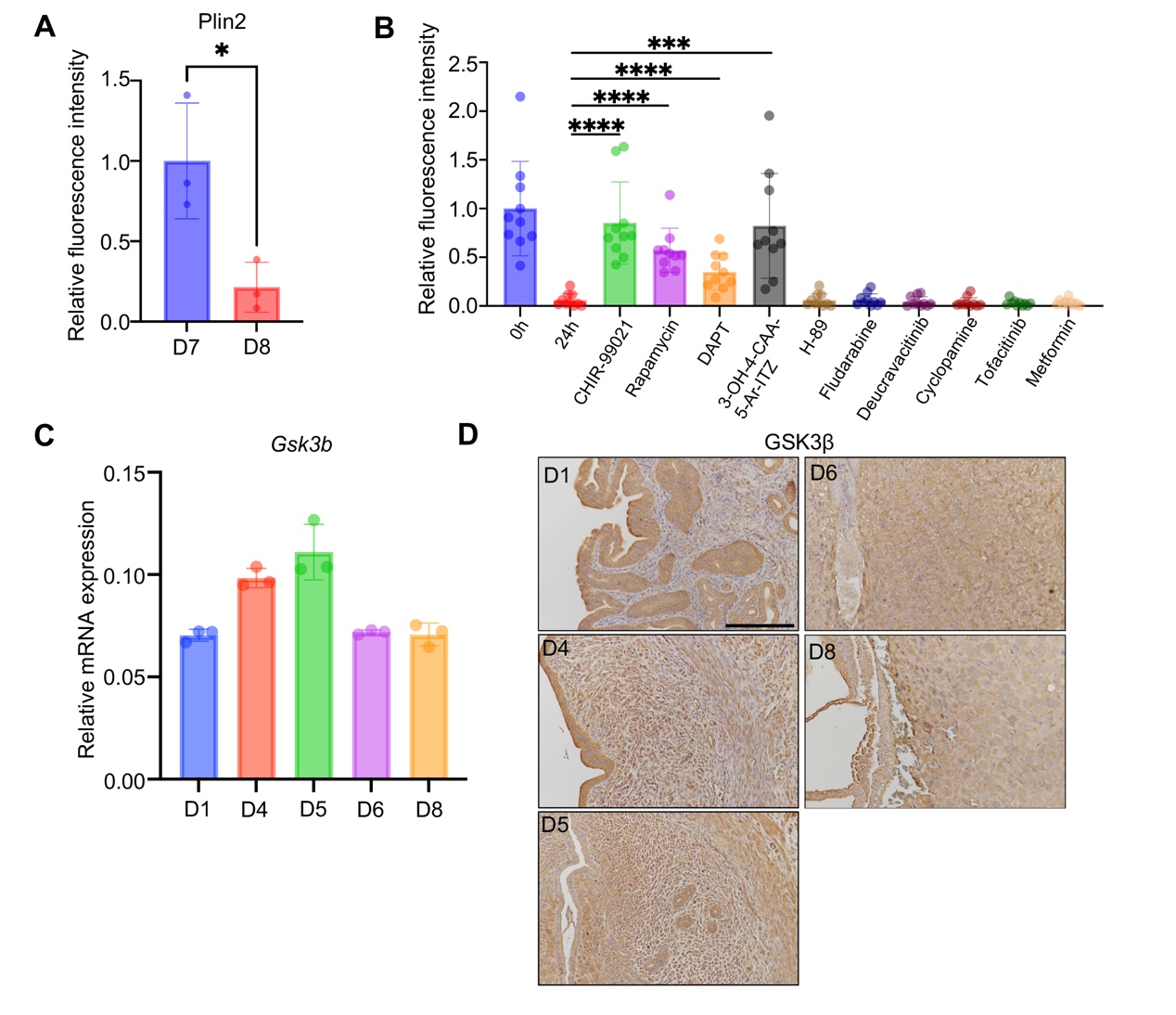
**

**Figure S1. GSK3β is highly expressed during decidualization.**

**A)** Quantification of data in Figure 1B (n = 3 animals), data represent the mean ± SEM. Two-tailed unpaired Student’s t-test, *p = 0.0257. **B)** Quantification of data in Figure 1F (n = 10 cells), data represent the mean ± SEM. Two-tailed unpaired Student’s t-test, ***p < 0.001, ****p < 0.0001. **C)** Quantitative real-time PCR analysis of *Gsk3b* mRNA levels in wildtype uteri (n = 3 animals) on days 1, 4, 5, 6, and 8 of pregnancy. The values are normalized to *Gapdh* and indicated as the mean ± SEM. **D)** Immunohistochemical analysis of GSK3β in wild type uteri on days 1, 4, 5, 6, and 8 of pregnancy. Scale bar: 200 μm.

**
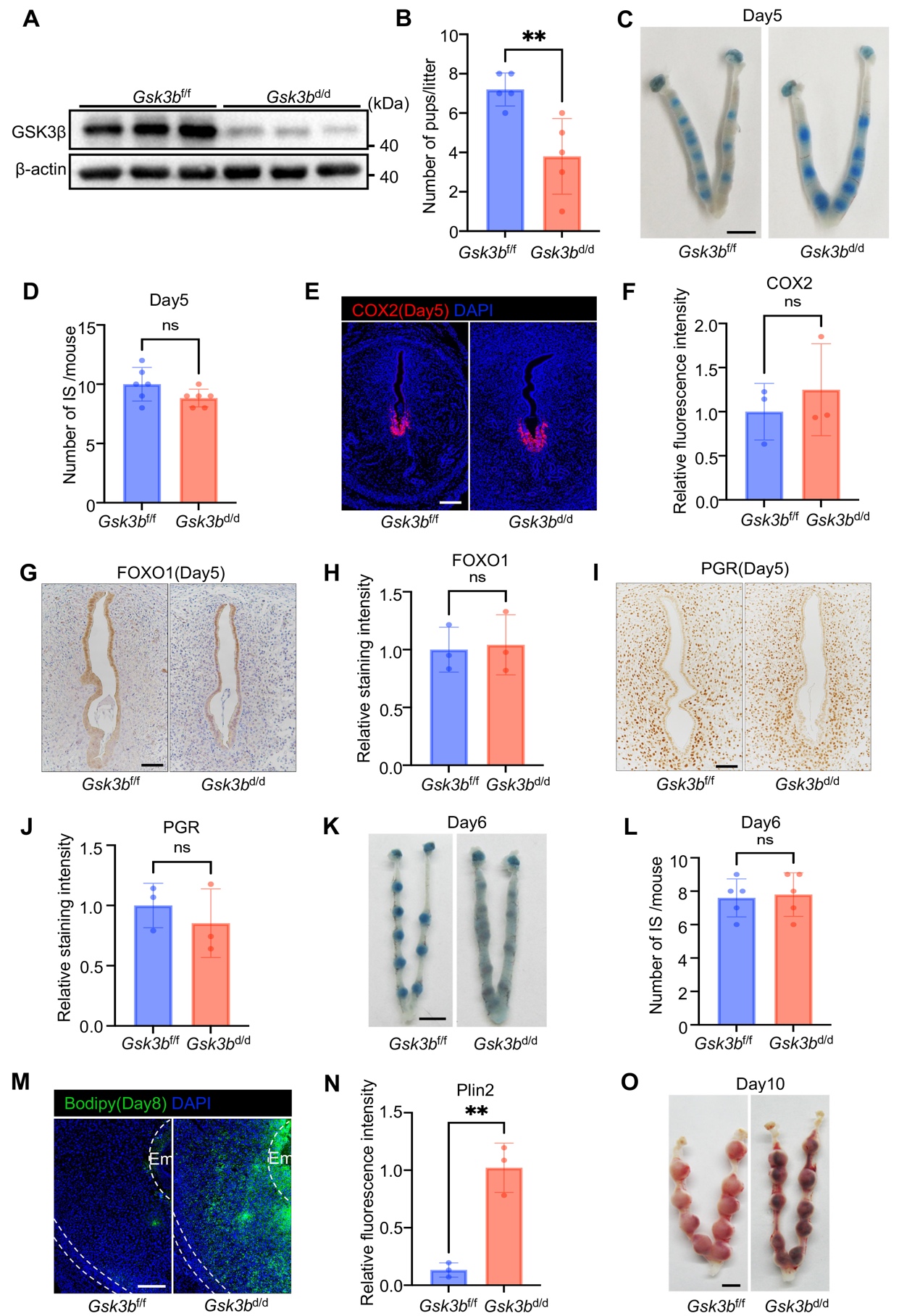
**

**Figure S2. Uterine *Gsk3b* ablation leads to impaired decidualization and miscarriage during mid-gestation.**

**A)** Immunoblot analysis of GSK3β in *Gsk3b*^f/f^ and *Gsk3b*^d/d^ uteri on day 4. **B)** Average litter sizes in *Gsk3b*^f/f^ (n = 5 animals) and *Gsk3b*^d/d^ (n = 5 animals) mice. Data represent the mean ± SEM. Two-tailed unpaired Student’s t-test, **p = 0.0067. **C**) Implantation sites marked by Chicago blue dye solution in *Gsk3b*^f/f^ and *Gsk3b*^d/d^ mice on day 5. Scale bar: 5 mm. **D)** The average number of implantation sites in *Gsk3b*^f/f^ (n = 6 animals) and *Gsk3b*^d/d^ (n = 6 animals) mice on day 5. Data represent the mean ± SEM. Two-tailed unpaired Student’s t-test. ns, not significant. **E)** Immunofluorescence analysis of embryo attachment reaction marker COX2 in *Gsk3b*^f/f^ and *Gsk3b*^d/d^ implantation sites on day 5. Scale bar: 200 μm. **F)** Quantification of data in Figure S2E (n = 3 animals), data represent the mean ± SEM. Two-tailed unpaired Student’s t-test, ns, not significant. **G)** Immunohistochemical analysis of FOXO1 in *Gsk3b*^f/f^ and *Gsk3b*^d/d^ implantation sites on day 5. Scale bars: 100 μm. **H)** Quantification of data in Figure S2G (n = 3 animals), data represent the mean ± SEM. Two-tailed unpaired Student’s t-test, ns, not significant. **I)** Immunohistochemical analysis of PGR in *Gsk3b*^f/f^ and *Gsk3b*^d/d^ implantation sites on day 5. Scale bars: 100 μm. **J)** Quantification of data in Figure S2I (n = 3 animals), data represent the mean ± SEM. Two-tailed unpaired Student’s t-test, ns, not significant. **K)** Implantation sites marked by Chicago blue dye solution in *Gsk3b*^f/f^ and *Gsk3b*^d/d^ mice on day 6. Scale bar: 5 mm. **L)** The average number of implantation sites in *Gsk3b*^f/f^ (n = 5 animals) and *Gsk3b*^d/d^ (n = 5 animals) mice on day 6. Data represent the mean ± SEM. Two-tailed unpaired Student’s t-test. ns, not significant. **M)** Bodipy fluorescent staining of lipid droplets in *Gsk3b*^f/f^ and *Gsk3b*^d/d^ uteri on day 8 of pregnancy. Scale bar: 200 μm. **N)** Quantification of data in Figure 2E (n = 3 animals), data represent the mean ± SEM. Two-tailed unpaired Student’s t-test, **p = 0.0023. **O)** Representative images of day 10 pregnant uteri in *Gsk3b*^f/f^ and *Gsk3b*^d/d^ mice. Scale bar: 5 mm.

**
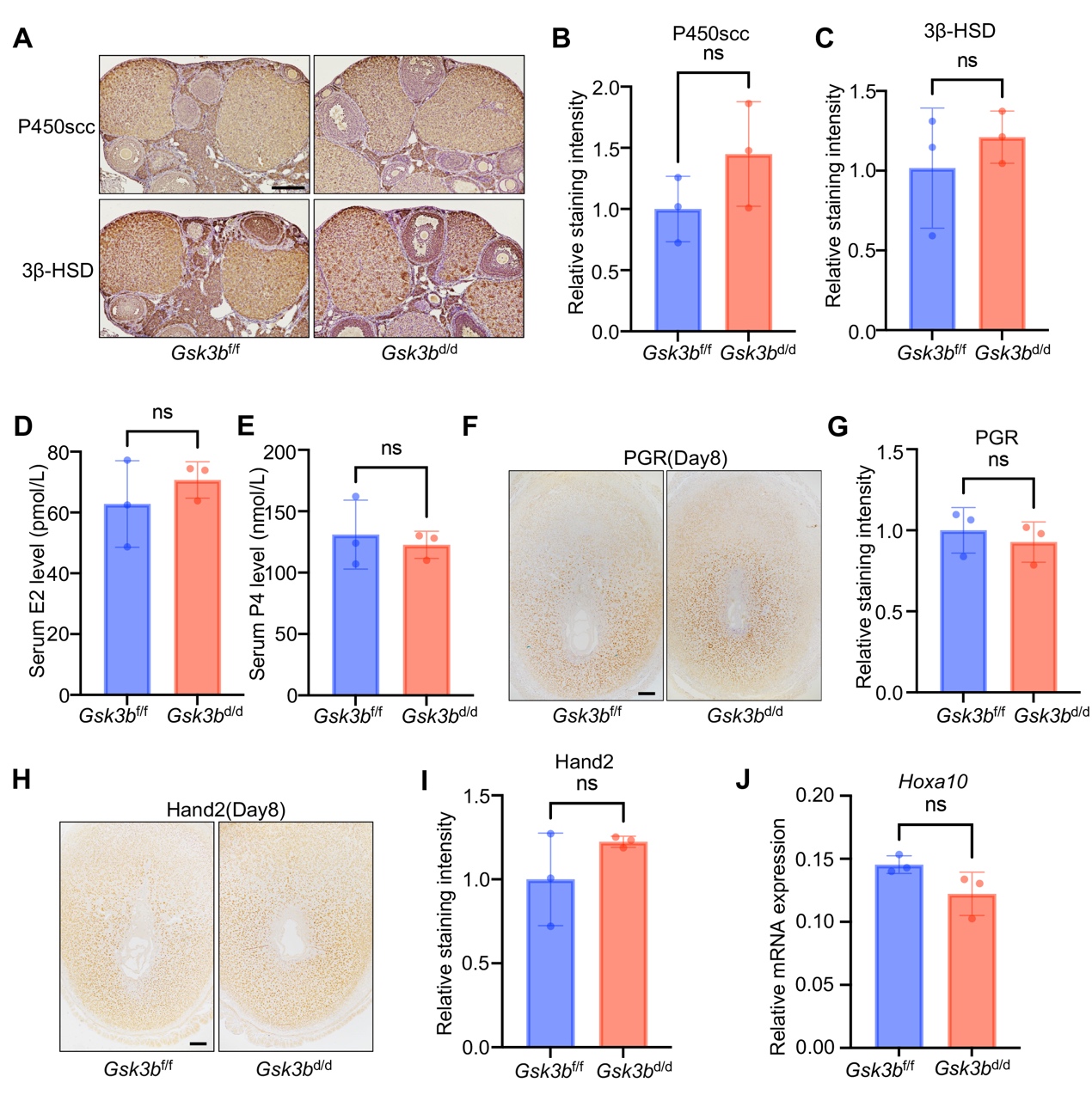
**

**Figure S3. Estrogen and progesterone level are comparable between the control and *Gsk3b* knockout mice.**

**A)** Immunohistochemistry staining of P450scc and 3β-HSD in day 8 *Gsk3b*^f/f^ and *Gsk3b*^d/d^ ovaries. Scale bar: 200 μm. **B-C)** Quantification of data in Figure S3A (n = 3 animals), data represent the mean ± SEM. Two-tailed unpaired Student’s t-test, ns, not significant. **D-E)** Serum estradiol-17β (E2) and progesterone (P4) levels in *Gsk3b*^f/f^ (n = 3 animals) and *Gsk3b*^d/d^ (n = 3 animals) mice on day 8. Data represent the mean ± SEM. Two-tailed unpaired Student’s t-test. ns, not significant. **F)** Immunohistochemical analysis of PGR in *Gsk3b*^f/f^ and *Gsk3b*^d/d^ implantation sites on day 8. Scale bars: 200 μm. **G)** Quantification of data in Figure S3F (n = 3 animals), data represent the mean ± SEM. Two-tailed unpaired Student’s t-test, ns, not significant. **H)** Immunohistochemical analysis of Hand2 in *Gsk3b*^f/f^ and *Gsk3b*^d/d^ implantation sites on day 8. Scale bars: 200 μm. **I)** Quantification of data in Figure S3H (n = 3 animals), data represent the mean ± SEM. Two-tailed unpaired Student’s t-test, ns, not significant. **J)** Quantitative real-time PCR analysis of *Hoxa10* mRNA levels in *Gsk3b*^f/f^ (n = 3 animals) and *Gsk3b*^d/d^ (n = 3 animals) implantation sites on day 8. The values are normalized to *Gapdh* and indicated as the mean ± SEM. Two-tailed unpaired Student’s t-test. ns, not significant.

**
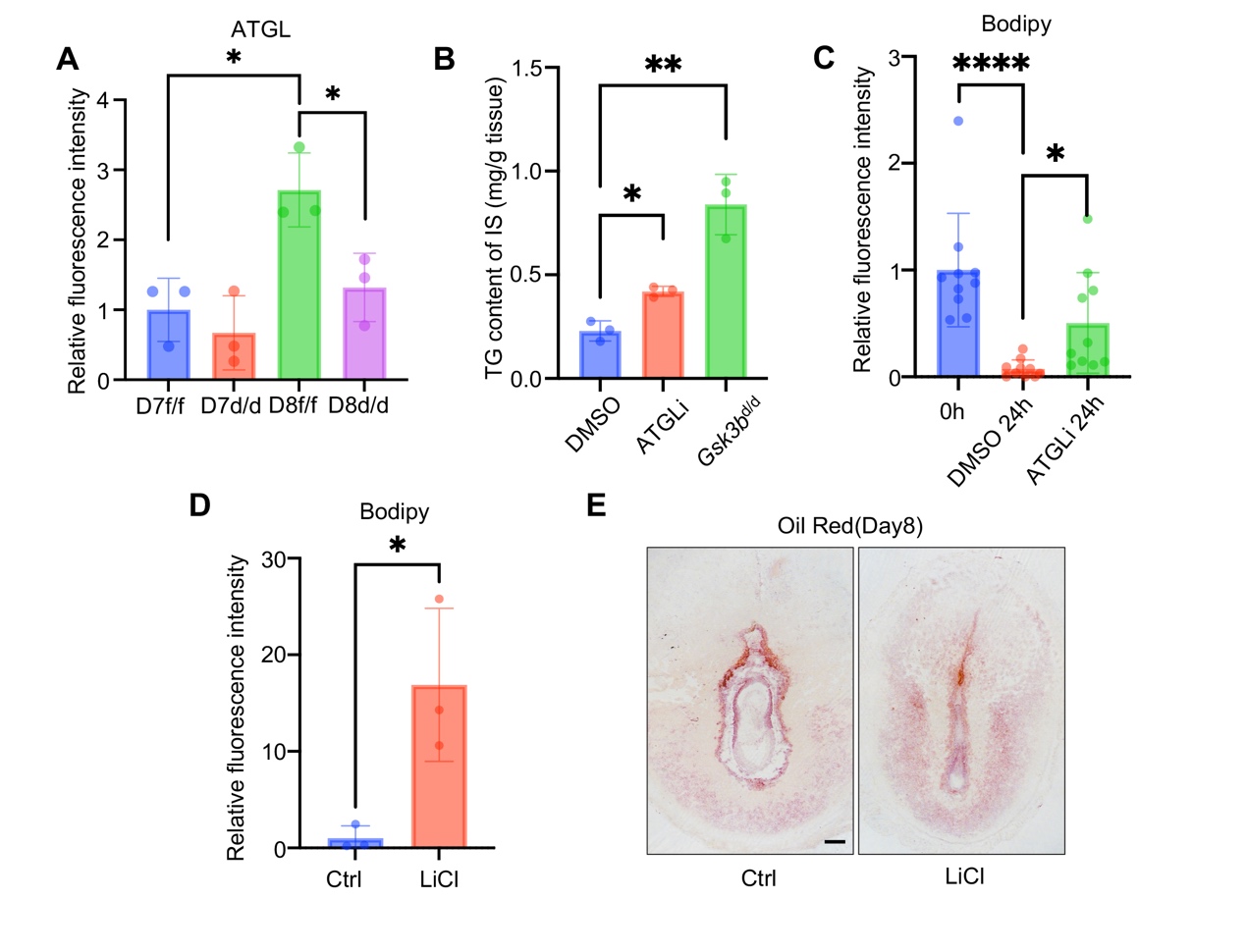
**

**Figure S4. GSK3 inhibitor LiCl treatment suppresses lipolysis during the decidualization.**

**A)** Quantification of data in Figure 4A (n = 3 animals), data represent the mean ± SEM. Two-tailed unpaired Student’s t-test. *p = 0.0131 (D8f/f vs D7f/f), *p = 0.0285 (D8d/d vs D8f/f). **B)** TG content assay of *Gsk3b*^f/f^  mice treated with DMSO or ATGLi and *Gsk3b*^d/d^ mice implantation sites on day 8 (n = 3 animals). Data represent the mean ± SEM. Two-tailed unpaired Student’s t-test, **p = 0.0037 (ATGLi), **p = 0.0023 (*Gsk3b*^d/d^). **C)** Quantification of data in Figure 4C (n = 10 cells), data represent the mean ± SEM. Two-tailed unpaired Student’s t-test. ****p < 0.0001, *p = 0.0109. **D)** Quantification of data in Figure 4D (n = 3 animals), data represent the mean ± SEM. Two-tailed unpaired Student’s t-test. *p = 0.0265. **E)** Oil red staining of lipid droplets in *Gsk3b*^f/f^ implantation sites treated with control or LiCl on day 8. Scale bars: 200 μm.


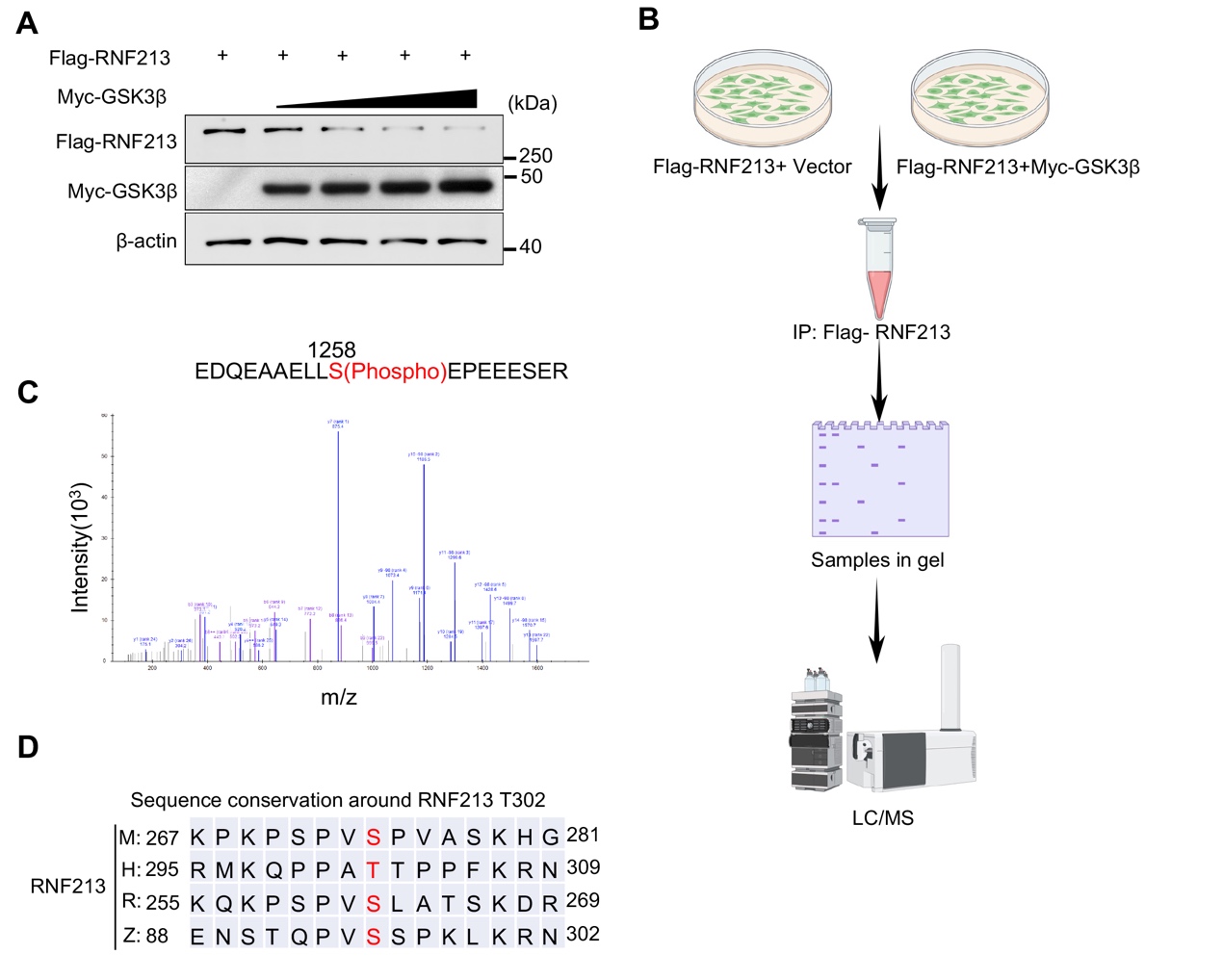


**Figure S5. GSK3β facilitates the degradation of RNF213 and regulates its phosphorylation.**

**A)** Western blot analysis showing Myc-GSK3β promotes the degradation of Flag-RNF213 in a dose-dependent manner. **B)** Schematic diagram illustrating the experimental approach for the identification of Flag-RNF213 phosphorylation site(s) catalyzed by Myc-GSK3β. **C)** Mass spectrometry profile of Flag-RNF213 peptide containing phosphorylated S1258. **D)** Amino acid sequence alignment showing sequence conservation of T302 and the adjacent region in RNF213 proteins from different species. M mouse, H human, R Rat and Z zebrafish.

**
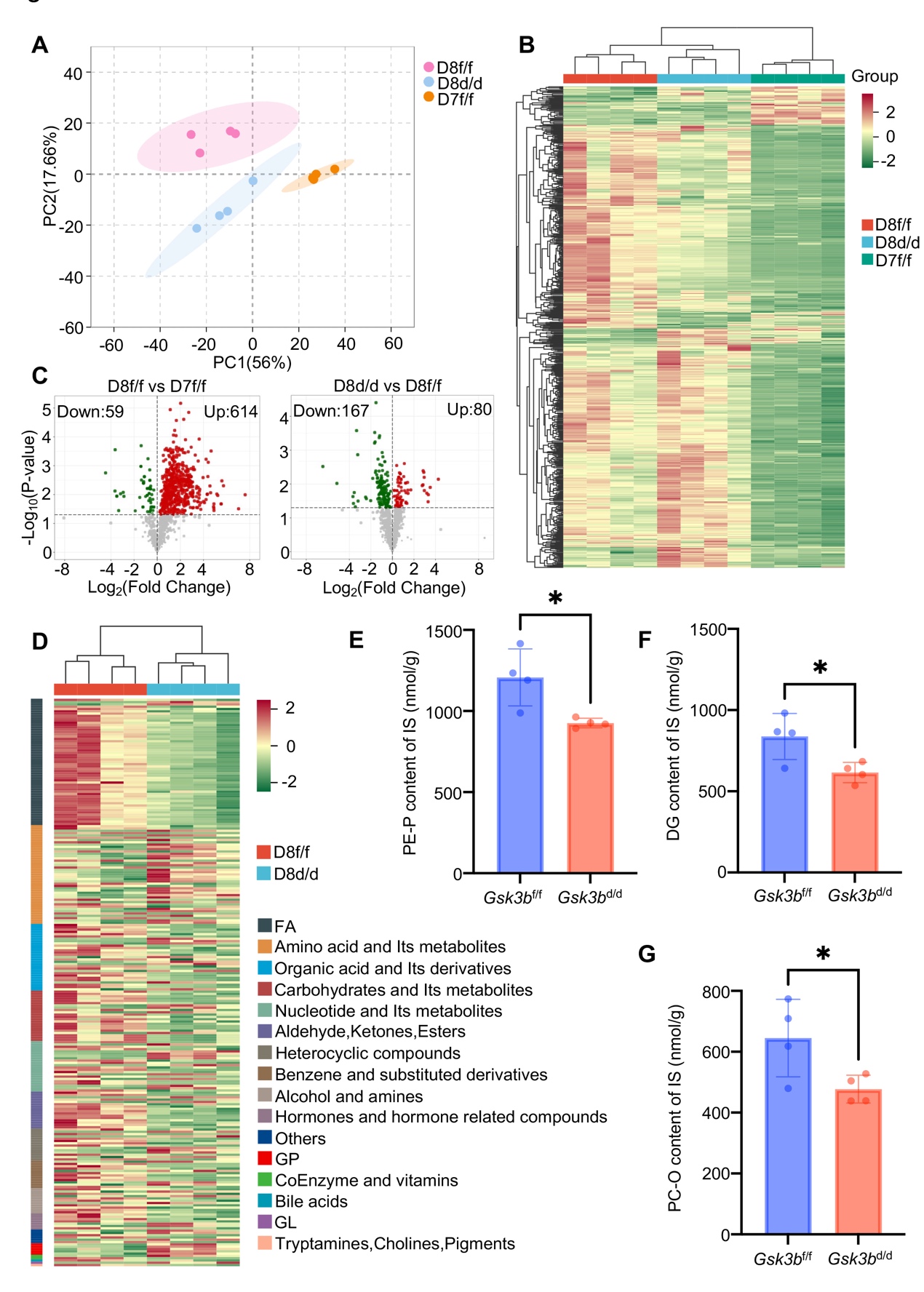
**

**Figure S6. *Gsk3b* deficiency primarily affects lipid composition rather than other metabolites.**

**A)** PCA plot of the metabolomics profiling data showing clustering of *Gsk3b*^f/f^ decidual tissues on day 7 and day 8 as well as *Gsk3b*^d/d^ decidual tissues on day 8. **B)** Heatmap showing the relative abundance of metabolomics between *Gsk3b*^f/f^ and *Gsk3b*^d/d^ decidual tissues on day 7 and day 8. **C)** Volcano plot showing the significantly changed metabolites between *Gsk3b*^f/f^ and *Gsk3b*^d/d^ decidual tissues on day 7 and day 8. **D)** Heatmap showing the relative abundance of lipid metabolomics between *Gsk3b*^f/f^ and *Gsk3b*^d/d^ decidual tissues on day 8. **E-G)** Bar graphs showing the levels PE-P, DG and PC-O in *Gsk3b*^f/f^ and *Gsk3b*^d/d^ decidual tissues on day 8. Data represent the mean ± SEM. Two-tailed unpaired Student’s t-test, *p = 0.0194 (PE-P), *p = 0.0286 (DG), *p = 0.0476 (PC-O).

**
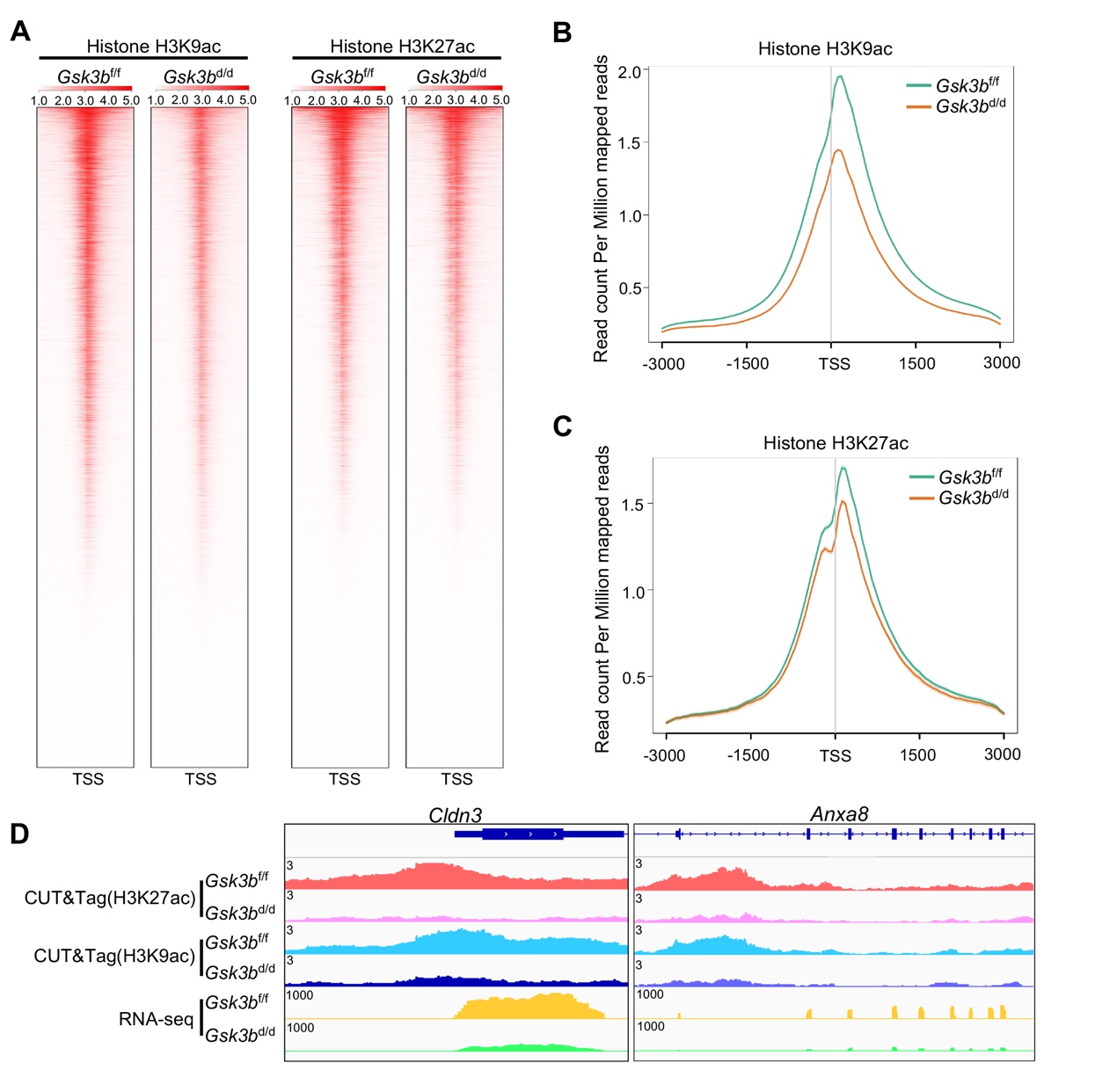
**

**Figure S7. Defective lipolysis results in reduced histone acetylation in *Gsk3b* knockout mice.**

**A)** Coverage profiles for histone H3K9ac and H3K27ac. Heatmap of peaks with decreased histone H3K9ac and H3K27ac following *Gsk3b* deletion centered at TSS. TSS, transcriptional start site. **B-C)** Profile plot showing the distribution of normalized H3K9ac and H3K27ac CUT&Tag signals. TSS, transcriptional start site. **D)** Genome browser view of normalized H3K9ac and H3K27ac CUT&Tag signals and RNA-seq tracks for representative genes, *Cldn3* and *Anxa8*.

**Table 1. Triglycerides with different side-chain lengths in Figure 1E**

| **1** | TG(14:0_16:0_16:0) | **40** | TG(16:0_18:2_18:2) | **79** | TG(18:0_18:2_22:4) |
| --- | --- | --- | --- | --- | --- |
| **2** | TG(14:0_16:1_17:1) | **41** | TG(16:0_18:2_20:4) | **80** | TG(18:0_18:2_22:6) |
| **3** | TG(14:0_16:1_18:1) | **42** | TG(16:0_18:2_22:6) | **81** | TG(18:0_18:3_20:2) |
| **4** | TG(14:0_18:0_18:2) | **43** | TG(16:0_20:0_18:1) | **82** | TG(18:0_20:4_22:6) |
| **5** | TG(14:0_18:0_20:0) | **44** | TG(16:0_20:0_22:6) | **83** | TG(18:0_22:5_22:6) |
| **6** | TG(14:0_20:2_22:6) | **45** | TG(16:0_20:1_22:0) | **84** | TG(18:1_18:1_18:1) |
| **7** | TG(16:0_16:0_16:0) | **46** | TG(16:0_20:2_22:6) | **85** | TG(18:1_18:1_18:2) |
| **8** | TG(16:0_16:0_16:1) | **47** | TG(16:0_20:4_24:6) | **86** | TG(18:1_18:1_20:1) |
| **9** | TG(16:0_16:0_18:0) | **48** | TG(16:0_24:0_18:1) | **87** | TG(18:1_18:1_22:1) |
| **10** | TG(16:0_16:0_18:1) | **49** | TG(16:1_16:1_18:1) | **88** | TG(18:1_18:1_22:2) |
| **11** | TG(16:0_16:0_20:4) | **50** | TG(16:1_16:1_24:6) | **89** | TG(18:1_18:1_22:4) |
| **12** | TG(16:0_16:0_22:6) | **51** | TG(16:1_18:0_20:1) | **90** | TG(18:1_18:1_24:4) |
| **13** | TG(16:0_16:0_24:0) | **52** | TG(16:1_18:1_18:1) | **91** | TG(18:1_18:2_18:2) |
| **14** | TG(16:0_16:1_16:1) | **53** | TG(16:1_18:1_18:2) | **92** | TG(18:1_18:2_18:3) |
| **15** | TG(16:0_16:1_18:1) | **54** | TG(16:1_18:1_18:3) | **93** | TG(18:1_18:2_20:0) |
| **16** | TG(16:0_16:1_18:2) | **55** | TG(16:1_18:1_22:1) | **94** | TG(18:1_18:2_20:1) |
| **17** | TG(16:0_16:1_20:1) | **56** | TG(17:0_17:0_19:1) | **95** | TG(18:1_18:2_20:2) |
| **18** | TG(16:0_16:1_20:4) | **57** | TG(17:0_18:0_18:1) | **96** | TG(18:1_18:2_20:4) |
| **19** | TG(16:0_16:1_22:6) | **58** | TG(17:0_18:1_18:2) | **97** | TG(18:1_18:2_22:0) |
| **20** | TG(16:0_17:1_18:1) | **59** | TG(17:1_18:1_18:1) | **98** | TG(18:1_18:2_22:3) |
| **21** | TG(16:0_18:0_18:0) | **60** | TG(18:0_16:1_20:1) | **99** | TG(18:1_18:2_22:6) |
| **22** | TG(16:0_18:0_18:1) | **61** | TG(18:0_16:1_24:6) | **100** | TG(18:1_20:1_20:1) |
| **23** | TG(16:0_18:0_18:2) | **62** | TG(18:0_16:2_20:2) | **101** | TG(18:1_20:1_22:1) |
| **24** | TG(16:0_18:0_20:0) | **63** | TG(18:0_18:0_18:0) | **102** | TG(18:1_20:1_22:4) |
| **25** | TG(16:0_18:0_20:1) | **64** | TG(18:0_18:0_18:1) | **103** | TG(18:1_20:2_18:3) |
| **26** | TG(16:0_18:0_22:0) | **65** | TG(18:0_18:1_18:1) | **104** | TG(18:1_20:2_20:4) |
| **27** | TG(16:0_18:1_18:1) | **66** | TG(18:0_18:1_18:2) | **105** | TG(18:1_20:3_22:4) |
| **28** | TG(16:0_18:1_18:2) | **67** | TG(18:0_18:1_20:0) | **106** | TG(18:1_22:1_18:2) |
| **29** | TG(16:0_18:1_20:1) | **68** | TG(18:0_18:1_20:1) | **107** | TG(18:1_24:1_18:2) |
| **30** | TG(16:0_18:1_20:2) | **69** | TG(18:0_18:1_20:3) | **108** | TG(18:2_18:2_20:1) |
| **31** | TG(16:0_18:1_20:4) | **70** | TG(18:0_18:1_20:4) | **109** | TG(18:2_18:2_22:1) |
| **32** | TG(16:0_18:1_22:0) | **71** | TG(18:0_18:1_22:3) | **110** | TG(18:2_18:2_22:6) |
| **33** | TG(16:0_18:1_22:1) | **72** | TG(18:0_18:1_22:4) | **111** | TG(18:2_20:3_20:4) |
| **34** | TG(16:0_18:1_22:3) | **73** | TG(18:0_18:1_22:5) | **112** | TG(18:2_22:5_22:6) |
| **35** | TG(16:0_18:1_22:4) | **74** | TG(18:0_18:1_22:6) | **113** | TG(20:0_18:1_22:6) |
| **36** | TG(16:0_18:1_22:5) | **75** | TG(18:0_18:2_20:0) | **114** | TG(20:1_18:2_18:0) |
| **37** | TG(16:0_18:1_22:6) | **76** | TG(18:0_18:2_20:2) | **115** | TG(20:1_18:2_22:6) |
| **38** | TG(16:0_18:1_24:1) | **77** | TG(18:0_18:2_20:3) |  |  |
| **39** | TG(16:0_18:1_24:4) | **78** | TG(18:0_18:2_20:4) |  |  |

**Table 2. The Hsc70-binding motifs within RNF213 were identified using the KFERQ Finder (version 0.8).**

| **Motif** | **Motif start** | **Motif type** |
| --- | --- | --- |
| KDLVK | 520 | acetyl. act. |
| KEVKR | 584 | acetyl. act. |
| QLREK | 1121 | canonical |
| LKKEK | 1150 | acetyl. act. |
| RFVTQ | 1689 | phos. act. |
| ELRKQ | 1715 | canonical |
| QYLRR | 2169 | phos. act. |
| KKLER | 2372 | acetyl. act. |
| KLERL | 2373 | acetyl. act. |
| QLVYR | 2563 | phos. act. |
| KLYIQ | 2591 | phos. act. |
| QRLVE | 2599 | canonical |
| RSLKQ | 2797 | phos. act. |
| QKSIV | 3184 | phos. act. |
| QVIER | 3232 | canonical |
| VIERQ | 3233 | canonical |
| VTRLQ | 3455 | phos. act. |
| KKFVD | 3737 | acetyl. act. |
| QRFRS | 3819 | phos. act. |
| RSRLQ | 3822 | phos. act. |
| KDVVK | 4308 | acetyl. act. |
| DVVKQ | 4309 | canonical |
| LVKDK | 4545 | acetyl. act. |
| QRRDV | 4563 | canonical |
| QDKRI | 4705 | canonical |
| QRDLV | 4791 | canonical |
| RDLVK | 4792 | acetyl. act. |
| DLVKQ | 4793 | canonical |
| LEKIQ | 4953 | canonical |
| QIVSR | 4959 | phos. act. |
| SRFLQ | 4962 | phos. act. |

**Table 3. Antibody information**

| **Antibodies** | **Company** | **Catalog No.** |
| --- | --- | --- |
| GSK3β | Cell Signaling Technology | 12456 |
| β-actin | Abmart | P30002 |
| FOXO1 | Cell Signaling Technology | 2880S |
| PGR | Cell Signaling Technology | 8757S |
| Plin2 | Proteintech | 15294-1-AP |
| RNF213 | Invitrogen | PA5-51902 |
| Flag | Sigma | F1804 |
| Myc | Abmart | M20002 |
| p-Ser | Santa Cruz | sc-81514 |
| p-Thr | Santa Cruz | sc-5267 |
| Ubiquitin | Cell Signaling Technology | 3936 |
| Hsc70 | Santa Cruz | sc-7298 |
| β-Catenin | Cell Signaling Technology | 8480 |
| Active-β-Catenin | Cell Signaling Technology | 19807 |
| H3K27ac | Active Motif | 39133 |
| H3K9ac | Cell Signaling Technology | 9649 |
| H3ac | Abcam | ab300641 |
| H3 | Proteintech | 17168-1-AP |
| ATGL | Abcam | ab207799 |
| P450scc | Santa Cruz | sc-18043 |
| 3β-HSD | Santa Cruz | sc-30820 |
| COX2 | Cell Signaling Technology | 12282S |
| Hand2 | Abcam | ab200040 |
| Dtprp | Homemade |  |
| Ki67 | Servicebio | GB121141 |
| pH3 | Cell Signaling Technology | 9701 |
| Claudin3 | Abcam | ab317319 |
| CD31 | BD | 553369 |

**Table 4. Inhibitor information**

| **Inhibitors** | **Company** | **Catalog No.** | **Target** | **Concentration** |
| --- | --- | --- | --- | --- |
| CHIR-99021 | MCE | HY-10182 | GSK3 | 3μM |
| Rapamycin | MCE | HY-10219 | mTOR | 10μM |
| DAPT | MCE | HY-13027 | Notch1 | 10μM |
| 3-OH-4-CAA-5-Ar-ITZ | MCE | HY-142042 | BMP2 | 10μM |
| H-89 | MCE | HY-15979 | PKA | 10μM |
| Fludarabine | MCE | HY-B0069 | STAT1 | 10μM |
| Deucravacitinib | MCE | HY-117287 | TYK2 | 10μM |
| Cyclopamine | MCE | HY-17024 | Hedgehog | 10μM |
| Tofacitinib | MCE | HY-40354 | JAK3/2/1 | 10μM |
| Metformin | MCE | HY-B0627 | AMPK | 10μM |
| Atglistatin | MCE | HY-15859 | ATGL | 20μM(Cell) 40mg/kg(Animal) |
| SKL2001 | MCE | HY-101085 | Wnt/β-catenin | 50mg/kg |
| Lithium chloride | MCE | HY-W094474 | GSK3 | 150 mg/kg |
| MG132 | MCE | HY-13259 | Proteasome | 10μM |
| CQ(Chloroquine) | MCE | HY-17589A | Lysosome | 25μM |
| CHX(Cycloheximide) | MCE | HY-12320 | Ribosome | 50 μg/mL |
