# Supplementary material for: GSK3β‐Regulated Lipolysis is Required for Histone Acetylation and Decidualization in Early Pregnancy
Source: Adv Sci (Weinh). 2025 Nov 9;13(5):e14291. doi: 10.1002/advs.202514291 (PMC12850330; doi:10.1002/advs.202514291)

Figure 1H

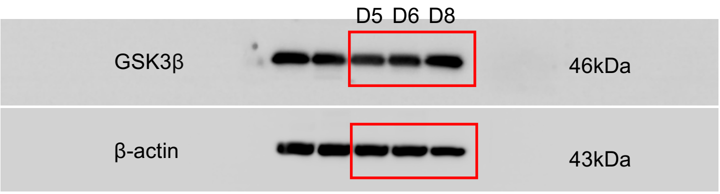

Figure S2A

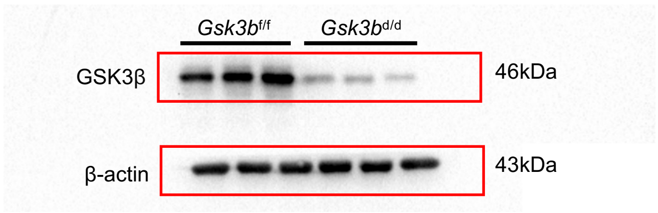

Figure 4E

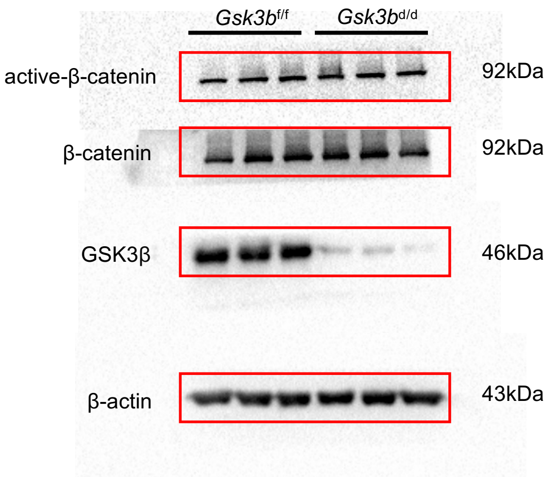

Figure 4F

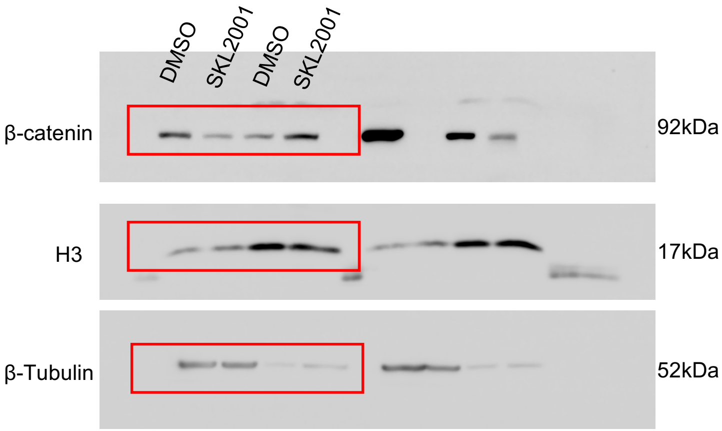

Figure 5D

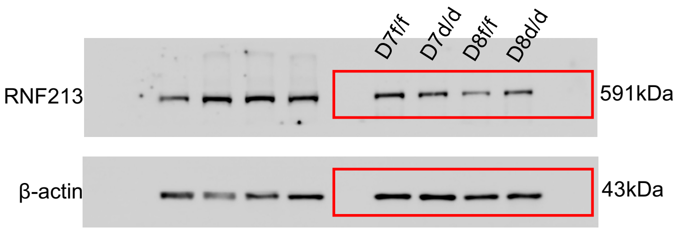

Figure 5E

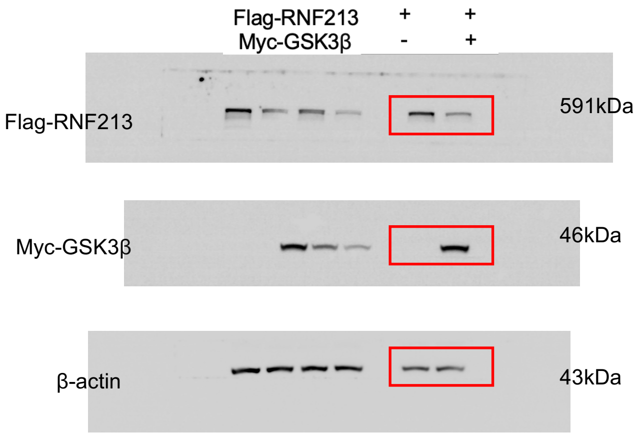

Figure 5G

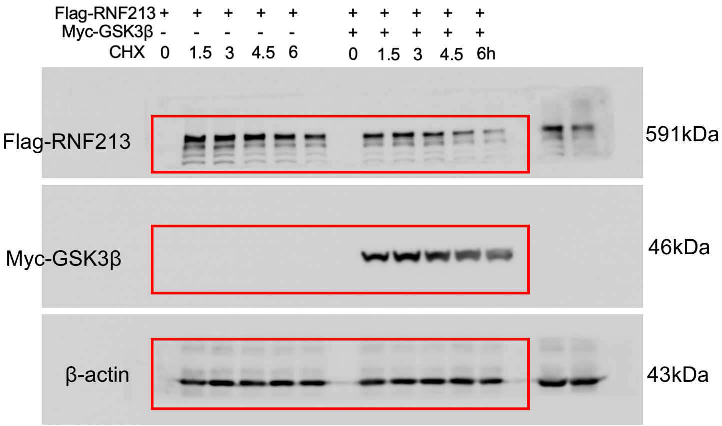

Figure 5H

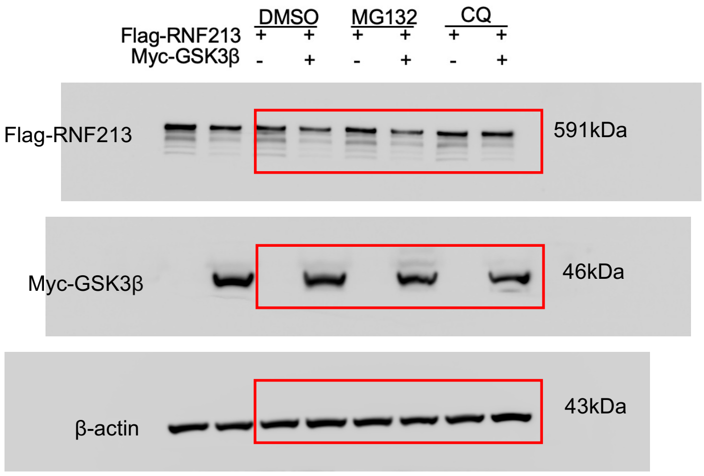

Figure 5SA

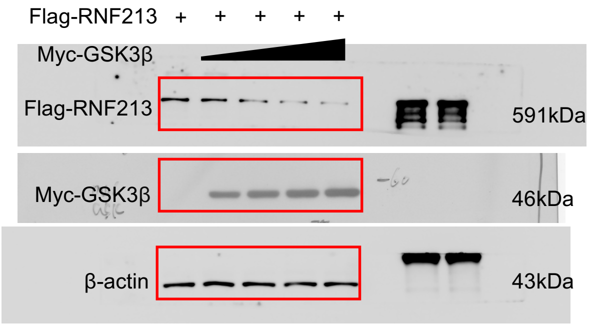

Figure 5I

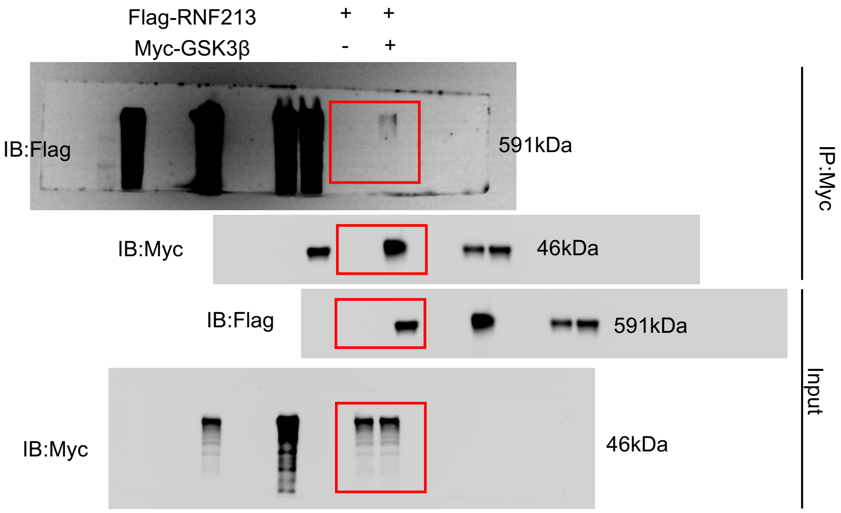

Figure 5J

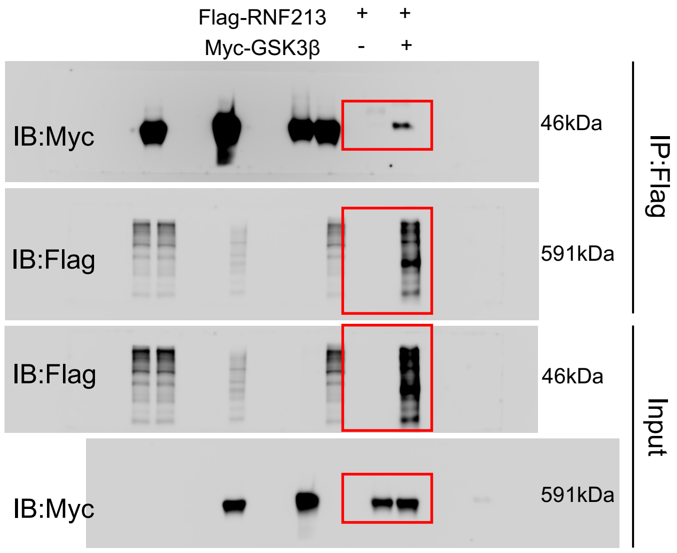

Figure 5K

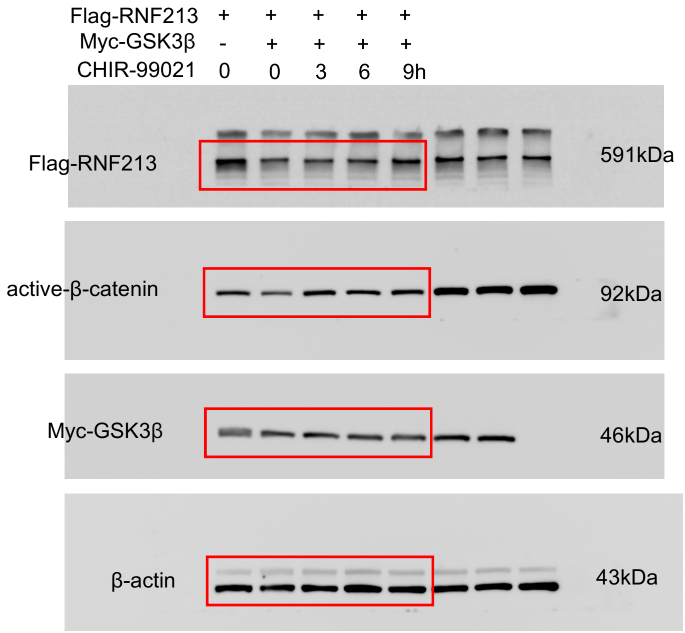

Figure 5L

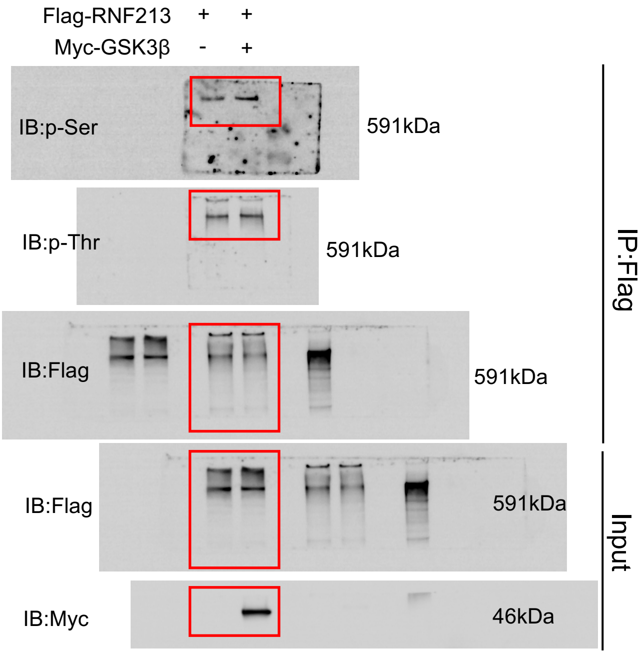

Figure 5N

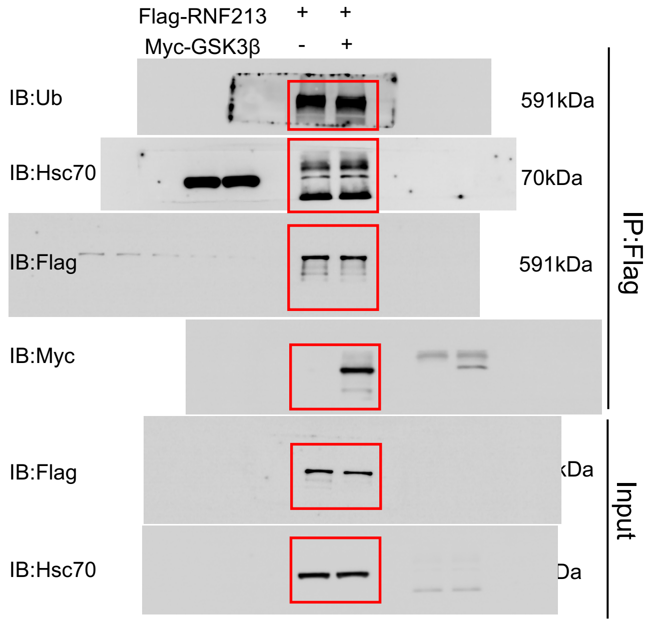

Figure 7C

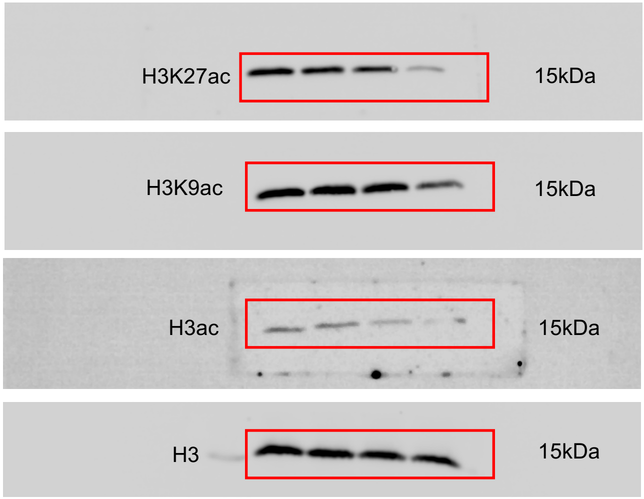

Figure 7K

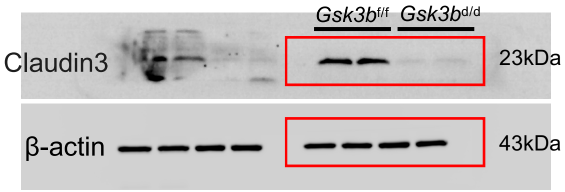

Supplement: Supplementary file 2 — Supporting Data File [file ADVS-13-e14291-s001.pdf]
